# A disulfidptosis-related glucose metabolism and immune response prognostic model revealing the immune microenvironment in lung adenocarcinoma

**DOI:** 10.3389/fimmu.2024.1398802

**Published:** 2024-07-18

**Authors:** Kai Zhang, Gang Li, Qin Wang, Xin Liu, Hong Chen, Fuqiang Li, Shuangyan Li, Xinmao Song, Yi Li

**Affiliations:** ^1^ Department of Oncology, 920th Hospital of Joint Logistics Support Force, Kunming, China; ^2^ Graduate School, Kunming Medical University, Kunming, China; ^3^ Department of Thoracic Surgery, Second Affiliated Hospital of Kunming Medical University, Kunming, China; ^4^ Department of Traditional Chinese Medicine, 920th Hospital of Joint Logistics Support Force, Kunming, China; ^5^ Department of Radiation Oncology, Ear, Nose & Throat Hospital of Fudan University, Shanghai, China

**Keywords:** lung adenocarcinoma, tumor microenvironment, disulfidptosis-related genes, immune checkpoint, programmed cell death

## Abstract

**Background:**

Lung adenocarcinoma accounts for the majority of lung cancer cases and impact survival rate of patients severely. Immunotherapy is an effective treatment for lung adenocarcinoma but is restricted by many factors including immune checkpoint expression and the inhibitory immune microenvironment. This study aimed to explore the immune microenvironment in lung adenocarcinoma via disulfidptosis.

**Methods:**

Public datasets of lung adenocarcinoma from the TCGA and GEO was adopted as the training and validation cohort. Based on the differences in the expression of disulfidptosis -related genes, a glucose metabolism and immune response prognostic model was constructed. The prognostic value and clinical relationship of the model were further explored. Immune-related analyses were performed according to CIBERSORT, ssGSEA, TIDE, IPS.

**Results:**

We verified that the model could accurately predict the survival expectancy of lung adenocarcinoma patients. Patients with lung adenocarcinoma and a low-risk score had better survival outcomes according to the model. Moreover, the high-risk group tended to have an immunosuppressive effect, as reflected by the immune cell components, phenotypes and functions. We also found that the clinically relevant immune checkpoint CTLA-4 was significantly higher in low-risk group (P<0.05), indicating that the high-risk group may suffer worse tumor immunotherapy efficacy. Finally, we found that this model has accurate predictive value for the efficacy of immune checkpoint blockade in non-small cell lung cancer (P<0.05).

**Conclusion:**

The prognostic model demonstrated the feasibility of predicting survival and immunotherapy efficacy via disulfidptosis-related genes and will facilitate the development of personalized anticancer therapy.

## Introduction

Lung cancer is one of the most common causes of cancer-related death worldwide, and lung adenocarcinoma (LUAD) accounts for the majority of lung cancer cases among all histological subtypes (1). Because LUAD is prone to metastasis in the early stage, the prognosis of LUAD patients is usually poor, with an average 5-year survival rate less than 20% (2). At present, personalized and precise treatments for lung cancer have been increasingly emphasized (3). Unfortunately, although great progress has been made in targeted therapy, the 5-year overall survival (OS) rate of LUAD patients is still low (4). Therefore, identification of better ways to improve the effectiveness of therapy is urgently needed.

Under normal circumstances, the immune system can identify and eliminate tumor cells in the tumor microenvironment (TME) (5). However, in order to survive and grow, tumor cells will escape the body’s immune surveillance in different ways, ultimately resulting in immune escape (6). Therefore, restoring the antitumor immune response to control and eliminate tumor cells is the core idea of ​​tumor immunotherapy (7). In clinical practice, immunotherapy has been successful at enhancing the tumor killing effect of tumor immune cells by inhibiting programmed death proteins (8). A case in point is pablizumab, whose inhibitory site is the PD-1 molecule. Tumor cells express PD-L1 and bind to the PD-1 receptor on effector T cells, thereby inducing programmed cell death (PCD) in effector T cells (9). PCD refers to the process in which cells initiate the expression of death-related genes through targeted signals in the internal and external environment, which promotes cell “suicide”, thus removing unnecessary or abnormal cells from the body (10). To date, the PCD family has expanded from apoptosis and necrosis to pyroptosis, ferroptosis, cuproptosis and other forms (11). In the latest research of Gan et al., a new form of PCD—disulfidptosis—was also found to be involved (12).

Disulfidptosis refers to glucose deficiency resulting in the excessive accumulation of disulfide bonds in cells highly expressing SLC7A11, which leads to abnormal crosslinking of disulfide bonds between cytoskeleton proteins (13), ultimately resulting in abnormal contraction of the cytoskeleton, collapse of the actin network and even cell death (14). Tumor cells usually need to highly express the SLC7A11 protein to recruit additional cystine for the synthesis of reduced glutathione, which balances the oxidation caused by the highly active metabolism of tumor cells (15). In addition, glucose metabolism plays an important role in the biochemical energy supply and cell substance transformation. Cystine entry into cells mediated by the SLC7A11 protein needs to be further reduced to cysteine by reduced nicotinamide adenine dinucleotide phosphate (NADPH) produced by the pentose phosphate pathway (PPP) in glucose metabolism (16). This process can reduce the toxicity of cystine and provide raw materials for the synthesis of glutathione. However, when glucose is deficient, NADPH depletion leads to abnormal accumulation of cystine and other disulfides in cells highly expressing SLC7A11 and triggers disulfidptosis (17). As a species of high-metabolism and high-energy-consumption cell, tumor cells with high SLC7A11 expression exhibit a stronger disulfidptosis response when glucose is depleted (18).

Therefore, in this study, we investigated disulfidptosis-related molecules and pathways using immune- and glucose metabolism-related genes by analyzing LUAD patient gene expression in the TCGA database. We established a risk model to predict LUAD patient survival and immunotherapy efficacy based on disulfidptosis-related genes, providing a therapeutic reference for LUAD patients.

## Methods

### Patients and datasets

The fragments per kilobase of transcript per million mapped reads (FPKM) standardized RNA-seq data of 600 samples, including 59 normal lung tissues and 541 tumor samples and corresponding clinical, prognostic and tumor mutation burden (TMB) data downloaded from The Cancer Genome Atlas (TCGA) website (https://portal.gdc.cancer.gov/projects/TCGA-LUAD), were used to identify DEGs between normal samples and tumor samples. Patients with unknown clinical information or an overall survival time less than 30 days were excluded. Then, three gene expression profiles of LUAD (GSE26939, GSE68465, and GSE72094) were downloaded from the Gene Expression Omnibus (GEO) (http://www.ncbi.nlm.nih.gov/geo) and used to validate the accuracy of the prognostic model. The 32 genomes involved in glucose metabolism were downloaded from the Molecular Signatures Database (MsigDB) via gene set enrichment analysis (GSEA; https://www.gsea-msigdb.org/gsea/msigdb/index.jsp) and used to identify disulfidptosis-related genes involved in glucose metabolism. Finally, two databases, IMMPORT (https://www.immport.org/) and InnateDB (https://www.innatedb.ca/), were used to obtain immune-related genes (19). The selected genes were subsequently used to identify disulfidptosis-related genes involved in the immune response.

### TCGA differential analysis

We performed differential expression analysis of genes encoding proteins (or their active subunits) that affect glucose metabolism in the TCGA cohort by the Wilcoxon test (20). Gene expression profiles were processed by CIBERSORT to determine the cell composition of complex tissues. The Wilcoxon test was subsequently used to analyze the difference in infiltrating immune cell diversity between normal tissues and lung adenocarcinoma tissues. The differential gene mechanism and signaling pathway enrichment analyses were performed by gene set variation analysis (GSVA) based on the Gene Ontology (GO) dataset (c5.go.v2023.1.Hs.symbols.gmt) and Kyoto Encyclopedia of Genes and Genomes (KEGG) dataset (c2.cp.kegg.v2023.1.Hs.symbols.gmt) from the TCGA cohort. We performed correlation analysis to identify disulfidptosis-related genes (DRGs) via the R packages “corrplot” and “circlize”. Additionally, heatmaps were constructed to visualize the results of the differential expression gene (DEG) analysis in the TCGA cohort via the R packages “limma” and “pheatmap”. Ultimately, immune cell infiltration was analyzed and visualized by correlation analysis between DEGs and immune cells.

### Identification of g/i-DRG-DEGs

A total of 24 DRGs were classified from recently published literature (21) to identify g/i-DRG-DEGs (disulfidptosis-related genes involved in glucose metabolism and the immune response). According to previous reports, Pearson analysis was considered an accepted method for revealing the correlation between DRGs and genes involved in glucose metabolism and the immune response in the RNA-seq data of TCGA LUAD patients (22). The cutoff values of R > 0.4 and P < 0.001 were acceptable. The differences in the expression levels of the g/i-Genes between LUAD tissues and normal tissues from the lungs were subsequently evaluated via the Wilcoxon test. A false discovery rate (FDR) < 0.05 and a fold change (FC)> 1 were set as screening criteria for obtaining differentially expressed g/i-DEGs. The g/i-DEG and g/i-DRGs intersect to obtain the g/i-DRG-DEG. The g-/i-DRG-DEGs were subsequently subjected to univariate Cox analysis to determine the prognostic value of the g-/i-DRG in LUAD patients via the R package “survival”. Least absolute shrinkage and selection operator (LASSO) Cox regression analysis was applied to construct a 7-g/i-DRG-DEG predictive signature (23).

### Construction of the disulfidptosis-related prognostic signature

Initially, 7 prognostic genes were screened out on the basis of the optimal penalty parameter l determined by tenfold cross-validation following the minimum criteria. Afterwards, Multivariate Cox regression analysis was conducted to establish a seven-gene predictive model. The computational formula used for determining the disulfidptosis-related prognostic risk score was as follows:

Risk score = Coefi gene1 × gene1 expression + Coefi gene2 × gene2 expression + ···· + Coefi gene × gene expression. Coefi represents the coefficient value of the corresponding gene. Patients were divided into low-risk and high-risk groups based on the median risk score (24).

Time-dependent receiver operating characteristic (ROC) analyses and Kaplan–Meier log-rank tests were used to evaluate the performance and prognostic ability of the predictive signature using the TCGA and GEO datasets via the R packages “timeROC” and “survival”, respectively. Additionally, univariate and multivariate Cox regression analyses were performed to assess the ability of the risk model to predict patient prognosis independent of other clinicopathological features.

### Clinical and functional analysis

A nomogram for predicting the 1-, 3-, and 5-year survival of LUAD patients was developed using the risk model in conjunction with clinicopathological parameters such as age, sex, and stage (25). We employed a calibration curve to determine if the anticipated survival rate was congruent with the observed survival rate. GSEA was performed to determine which pathway genes were enriched mainly between the high- and low-risk groups via the GO dataset and the KEGG dataset from the molecular signature dataset (https://www.gsea-msigdb.org/gsea/msigdb) as references. The criteria for statistical significance were FC>1, nominal p<0.05 and FDR<0.25. Then, functional enrichment analyses based on the KEGG dataset and GO dataset were performed separately (26).

### Immune infiltration analysis

Twenty-nine different kinds of tumor infiltrating immune cells (TIICs) were examined by ssGSEA (27). The Wilcoxon test was performed to compare the tumor-infiltrating immune cell scores. K−M survival curves and the log-rank test were used to compare the prognostic significance of immune cells with significant differences between the high-infiltration and low-infiltration groups in the TCGA cohort (28). Additionally, the expression levels of immune checkpoint molecules were extracted from 541 LUAD tissues in the TCGA database. The differential expression of immune checkpoint molecules in the high- and low-risk groups was explored using the Wilcoxon test. The same method was used to analyze the difference in HLA expression (29).

### Immune analysis

Tumor immune dysfunction and exclusion (TIDE) is a computational framework for evaluating the possibility of tumor immune escape according to the gene expression profiles of tumor samples. We obtained TIDE scores (http://tide.dfci.harvard.edu/) and performed a difference analysis of TIDE scores for the high- and low-risk groups using Wilcox’s method to predict immune escape. To further verify the accuracy of the risk score model, we generated ROC curves for the risk score, TIDE score and TIS. First, we calculated the tumor mutation burden (TMB) for each tumor sample in the TCGA cohort and performed differential analysis of the TMB using the Wilcoxon test. Subsequently, we performed combined survival analysis on the same samples by combining TMB and risk score data. We classified tumors into six subtypes based on immunological characteristics (30). The immune checkpoint inhibitor (ICI) sensitivity score of tumor samples from each TCGA cohort was calculated by the R package “oncoPredict”. Finally, we downloaded the immune cell proportion score (IPS) from The Cancer Imaging Archive database (TCIA) and combined it with the TCGA expression data. We used the R packages reshape2 and ggpubr to perform a rank sum test on the IPS between the high- and low-risk groups, and the results were visualized with box plots.

### Other statistical analyses

RStudio and its associated packages were used to conduct all the statistical analyses. The ‘ggplot2’ package was used to visualize the graphs. Wilcox analysis was performed through the ‘limma’ package. The chi-square test was used to examine differences in the proportions of clinical features. A paired t test was used to analyze the difference in the survival of LUAD tissues and adjacent normal tissues. Differences among multiple groups were analyzed by one-way ANOVA. p<0.05 was considered to indicate statistical significance.

## Results

### Selection and differential analysis of the TCGA cohort

**Figure 1:**
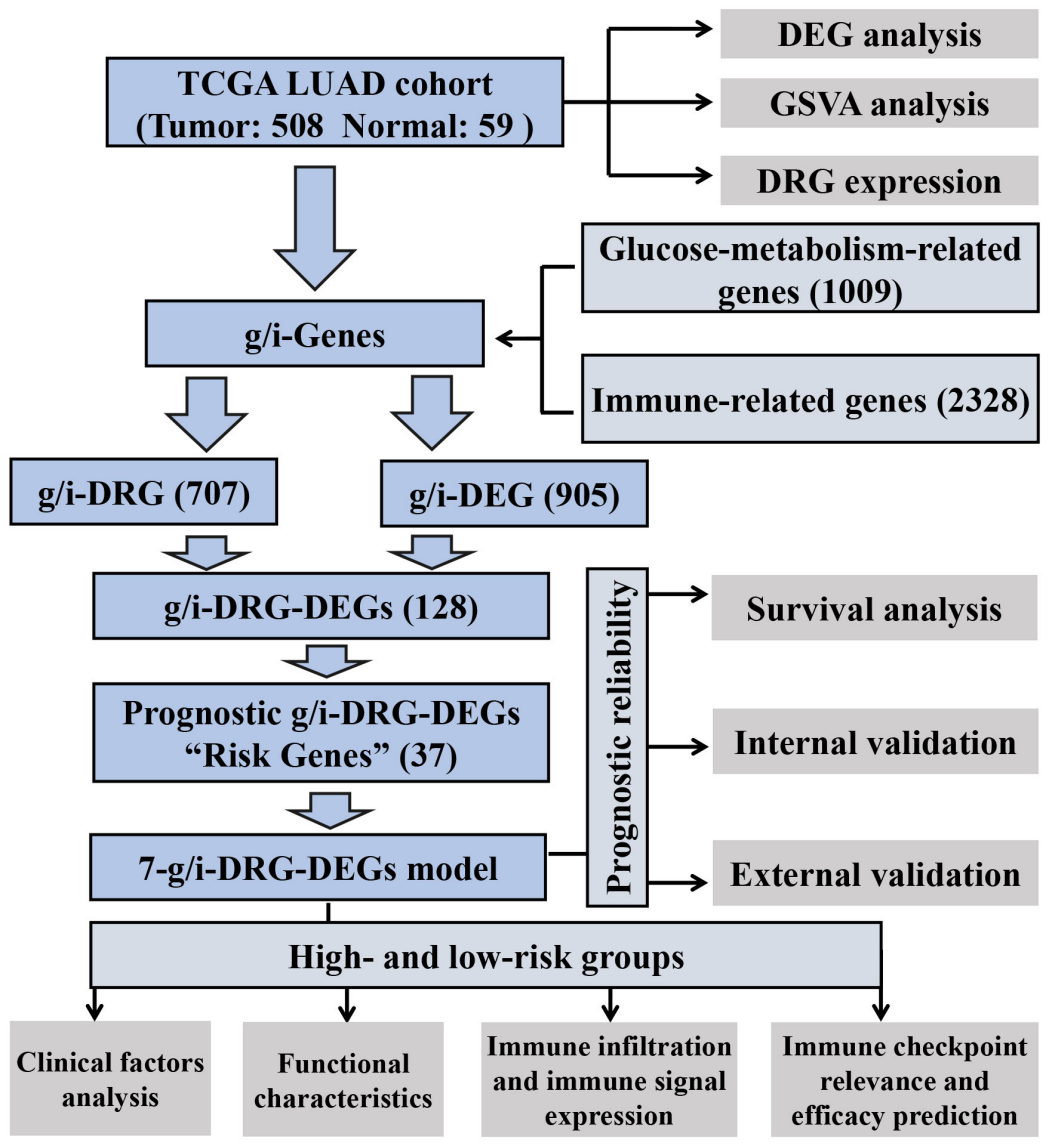
Analysis workflow of our work. LUAD, Lung Adenocarcinoma; TCGA, The Cancer Genome Atlas; GSVA, Gene Set Variation Analysis; DEG, Differential Expression Gene; DRG, Disulfidptosis-Related Gene.

**Figure 2:**
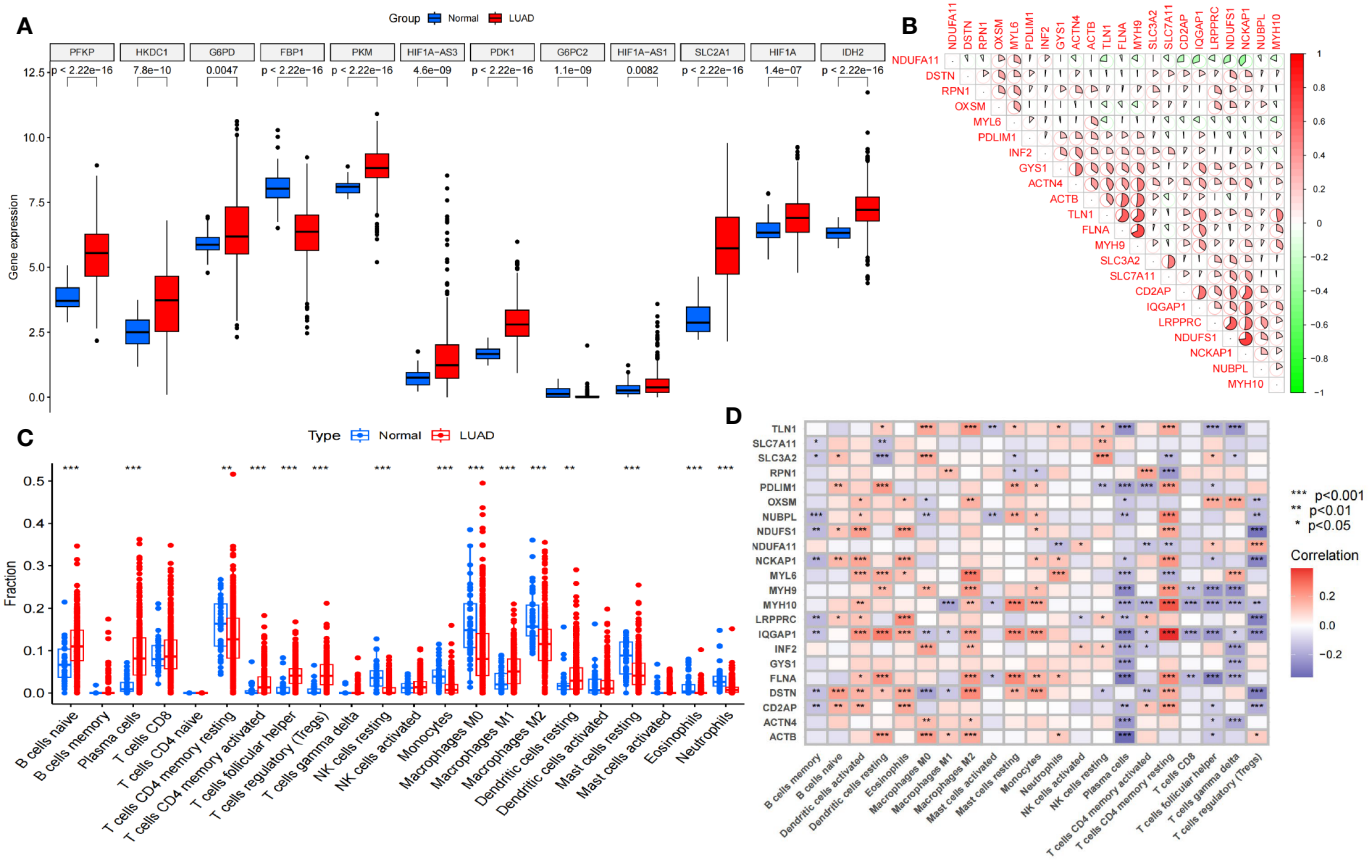
Selection and difference analysis of the TCGA cohort. **(A)** Differential expression analysis of the key glycolysis-related genes involved in disulfidptosis. **(B)** Correlation analysis of 22 disulfidptosis-related genes expressed in this TCGA cohort. **(C)** Analysis of immune cells in the tumor microenvironment in this TCGA cohort. **(D)** Disulfidptosis-related genes correlation analysis with different tumor-infiltrating immune cells. NK cells, Natural Killer cells. *P<0.05; **P<0.01; *** P<0.001.

The overall data analysis workflow is shown in **Figure 1**. We selected the TCGA cohort, which included 600 samples (including 541 LUAD cases and 59 normal tissue cases). Samples with duplicate names were removed by quality control (the expression values of the same patient were averaged), and 508 LUAD samples were ultimately obtained. We annotated the standardized RNA-seq data in the TCGA cohort and obtained a total of 59427 genes. By analyzing the differential expression of the genes affecting glucose metabolism, we found that several hypoxia-inducible genes (such as HIF-1A and PDK-1, P<0.05) were significantly upregulated. Moreover, glucose transport-, glycolysis-, and pentose phosphate pathway-related genes (such as SLC2A1, PKM, PFKP, IDH2 and G6PDH, P<0.05) were significantly upregulated, suggesting that there were additional active glucose transport, glycolysis and pentose phosphate pathway processes in LUAD cells (**Figure 2A**).

Furthermore, we analyzed the differences in immune cells in the tumor microenvironment in this cohort (**Figure 2C**). Through gene set variation analysis (GSVA) analysis based on Kyoto Encyclopedia of Genes and Genomes (KEGG) and Gene Ontology (GO) analyses, we found significant differences in immune-related, metabolism-related and tumor-related pathways (**Supplementary Figures S1A, B**). Moreover, the expression of 24 disulfidptosis-related genes (DRGs) was significantly different in different tumor-infiltrating immune cells (**Figure 2D**), and most DRGs were expressed at low levels in CD4+ regulatory T cells and plasma cells. We found that there were 22 DRGs in the TCGA cohort (**Figure 2B**). We performed differential expression analysis of the DRGs, and all the DRGs were significantly different between normal and tumor tissues (P<0.05). Among them, SLC7A11, LRPPRC and other genes were significantly expressed at low levels in normal tissues, while MYH10, PDLIM1 and other genes were significantly highly expressed in normal tissues (**Supplementary Figure S1C**).

### Identification and construction of the 7-g/i-DRG-DEG signature model

**Figure 3:**
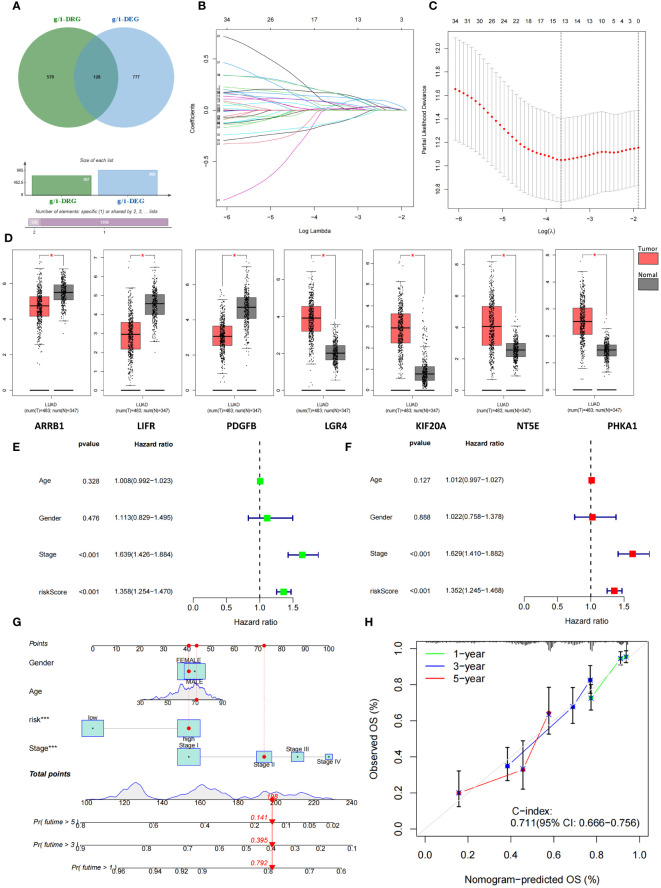
Identification and construction of 7-g/i-DRG-DEG signature model. **(A)** Combining two gene sets to obtain the total gene set (g/i-Genes) related to glucose metabolism or the immune response. **(B, C)** Cvfit **(B)** and lambda curves **(C)** demonstrating the generated LASSO regression of 7 signature genes. **(D)** Differential expression of 7 signature genes between tumor and normal tissue. **(E, F)** Univariate **(E)** and multivariate Cox regression analyses **(F)** of overall survival in LUAD patients. **(G, H)** Nomogram analysis **(G)** and calibration curve **(H)** predicting LUAD patient prognosis. *P<0.05.

We selected a total of 1009 genes from 32 glucose metabolism-related gene sets from the GSEA database and then extracted the expression of 1009 genes from the TCGA cohort via Perl scripts. Using the same method, we evaluated the expression of 2328 genes involved in the immune response from the IMMORT and INNATE gene sets. These two gene sets were combined to obtain the total gene set (g/i-Genes) related to glucose metabolism or the immune response (**Figure 3A**). The expression levels of the g/i-Genes were performed via differential gene analysis and correlation analysis with the DRGs: *a*. Through differential gene expression analysis (log2|FC|>1, FDR<0.01), we obtained 905 glucose metabolism- and immune-related genes (g/i-DEGs). *b*. A total of 707 DRG-related genes (g/i-DRGs) were screened by Pearson correlation analysis (R > 0.4, P < 0.001). A total of 128 differentially expressed disulfidptosis-related genes involved in glucose metabolism and the immune response (g/i-DRG-DEG) were obtained by taking the intersection of g/i-DEG and g/i-DRG. Ultimately, we used the overall survival (OS) data of LUAD patients in the TCGA cohort to examine the predictive ability of g/i-DRG-DEGs through univariate Cox regression analysis, and 37 prognostic g/i-DRG-DEGs were identified as “risk genes”.

Subsequently, 508 LUAD patients were randomly divided into two groups: the training group and the validation group. We used the expression profiles of 37 prognostic g/i-DRG-DEGs in the training cohort to construct a 7-g/i-DRG-DEG model containing 7 signatures through LASSOCOX regression analysis, including ARRB1、LIFR、PDGFB、LGR4、KIF20A、NT5E、PHKA1 (**Figures 3B, C**). There were significant differences in the expression of these seven genes in the TCGA cohort (**Figure 3D**). Multivariate Cox regression was used to analyze the expression risk score of the 7-g/i-DRG-DEG for each sample. Tumor stage and risk score were found to be important predictors of OS in LUAD patients by univariate Cox regression analysis (**Figure 3E**, P<0.001). Tumor stage and the risk score were independent determinants of OS in LUAD patients by multivariate Cox analysis (**Figure 3F**, P<0.001). To better demonstrate the prognostic value of this model for LUAD patients, we used a nomogram to predict the prognosis of LUAD patients at 1, 3, and 5 years (**Figure 3G**). The calibration curve further verified that the 1-, 3-, and 5-year survival rates were highly consistent with the predicted survival rates (**Figure 3H**).

### Prognostic reliability of 7-g/i-DRG-DEG model

**Figure 4:**
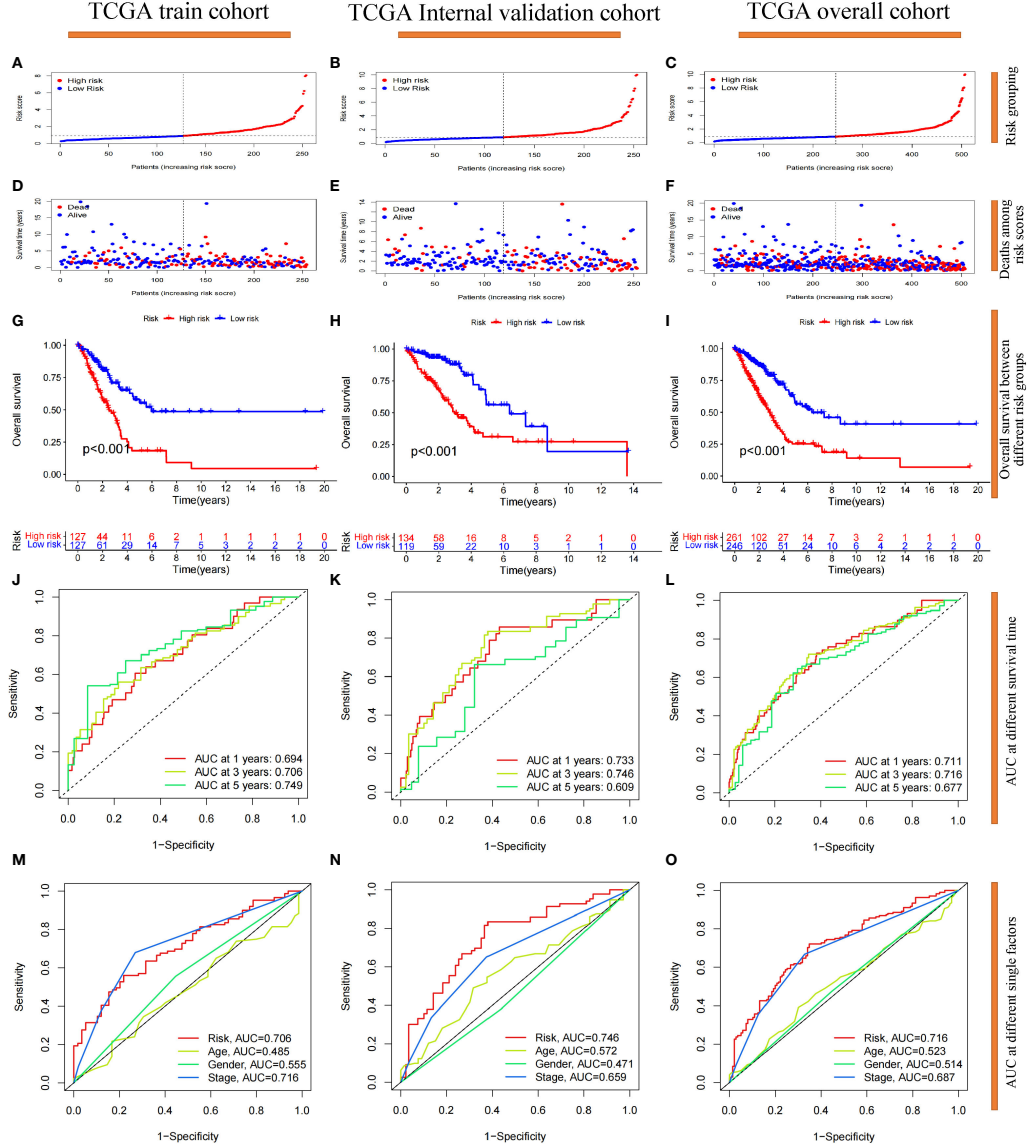
Prognostic reliability validation of the 7-g/i-DRG-DEG signature model. **(A–C)** Dividing the cohort into high- and low-risk groups by the median cutoff. **(D–F)** Deaths situation of LUAD patients in the cohort between high- and low-risk groups. **(G–I)** Overall Survival of LUAD patients between the high- and low-risk groups. **(J–L)** Area Under Curve (AUC) at 1-year, 3-year and 5-year survival time. **(M–O)** AUC at risk group, age, gender and tumor stage. **(A, D, G, J, M)** Analysis of the TCGA training cohort. **(B, E, H, K, N)** Analysis of the TCGA internal validation cohort. **(C, F, I, L, O)** Analysis of the TCGA entire cohort.

We focused on the prognostic value of the 7-g/i-DRG-DEG signature model and evaluated its performance. Patients in the TCGA training cohort were divided into high-risk and low-risk groups by the median cutoff (**Figure 4A**), and deaths among LUAD patients increased as risk scores increased (**Figure 4D**). K−M curve analysis revealed that OS was significantly shorter in the high-risk subgroup than in the low-risk subgroup (P<0.001) (**Figure 4G**). Moreover, the area under the curve (AUC) values for 1-year, 3-year and 5-year survival were 0.694, 0.706 and 0.749, respectively (**Figure 4J**). The risk score had greater predictive accuracy than did the other single factors (**Figure 4M**). To evaluate the prognostic value of OS in the entire TCGA dataset, we further performed confirmatory analyses of the model in the validation cohort and the entire TCGA cohort. Consistent with the results observed in the training cohort, samples from both risk categories were reasonably distributed in the validation cohort (**Figures 4B, E, H, K, N**) and the entire cohort (**Figures 4C, F, I, L, O**). Finally, we conducted external validation on three GEO datasets (the GSE26939, GSE68465 and GSE72094 cohorts) to further verify the generalizability of the model. The results demonstrated that the model had the same stable performance (**Supplementary Figure S2**). The above analyses revealed that the disulfidptosis-related 7-g/i-DRG-DEG signature is a reliable independent predictor of LUAD patients.

### The clinical and functional characteristics of risk score based on the 7-g/i-DRG-DEG model

First, we visualized the DEGs between the high-risk and low-risk groups via heatmaps (**Supplementary Figure S3**). A total of 782 genes were significantly differentially expressed. Among the seven modeling genes, NT5E (log2|FC|=1.06, p<0.05) and KIF20A (|FC|=1.02, p<0.05) were significantly highly expressed in the high-risk group. We divided LUAD patients in the TCGA cohort into different groups randomly according to clinical stage, T stage, age, sex, and four representative gene mutations (KRAS, EGFR, TP53, STK11) to study whether the prognostic model could predict LUAD patient OS based on these clinical features (**Supplementary Figure S4**). We also performed a correlation analysis of risk scores between different clinical variable subgroups and detected significant differences in tumor stage (P<0.001), T stage (P<0.05) and TP53 mutation status (P<0.001). In addition, we found that the OS time of high-risk patients was significantly shorter than that of low-risk patients in every clinical characteristic subgroup (P<0.01). In summary, the 7-g/i-DRG-DEG model can accurately predict the prognosis of LUAD patients without considering certain essential clinical characteristics.

**Figure 5:**
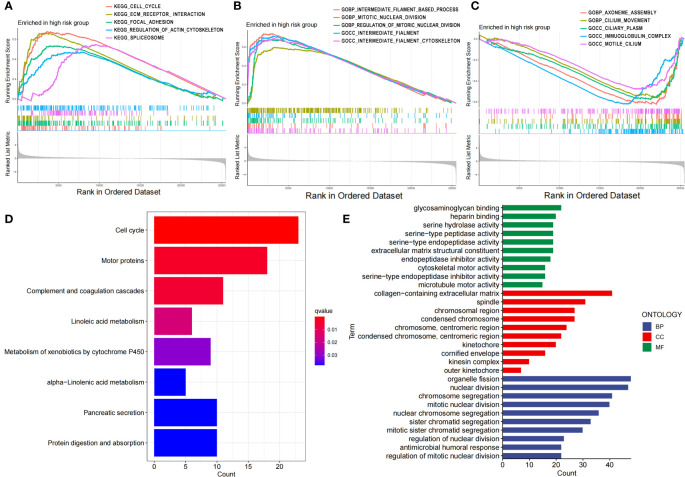
Functional characteristics of the risk score based on the 7-g/i-DRG-DEG model. **(A–C)** GSEA analyses between the high- and low-risk groups. **(D, E)** KEGG **(D)** and GO **(E)** analyses between the high- and low-risk groups.

Then, we performed GSEA, KEGG and GO enrichment analyses. Through gene set enrichment analysis (GSEA), we found that several pathways related to tumor development and progression, including the cell cycle, ECM receptor interaction, focal adhesion, actin regulatory, and spliceosome pathways, were upregulated in the high-risk group (**Figure 5A**), wherein actin regulation is closely related to disulfide denaturation. Moreover, there were also significant differences in several biological processes between the two groups, including intermediate filament, mitotic nuclear, mitotic nuclear regulation, axial filament assembly and ciliary movement (**Figures 5B, C**). KEGG enrichment analysis revealed significant differences in the cell cycle, motor proteins, complement and coagulation cascades, pancreatic secretion, protein digestion and absorption, metabolism of xenobiotics by cytochrome P450, linoleic acid metabolism and the alpha−linolenic acid metabolism pathway (**Figure 5D**). GO functional enrichment analysis also revealed significant differences in cytoskeletal motor activity, microtubule motor activity, glycosaminoglycan binding, heparin binding, serine metabolism-related enzyme activities, organelle fission, nuclear division, chromosome segregation, chromosome-associated regions, organelles involved in cell division, and the collagen-containing extracellular matrix (**Figure 5E**). Importantly, cytoskeletal motor activity and microtubule motor activity are strongly correlated with disulfidptosis.

### Immune correlation analysis

**Figure 6:**
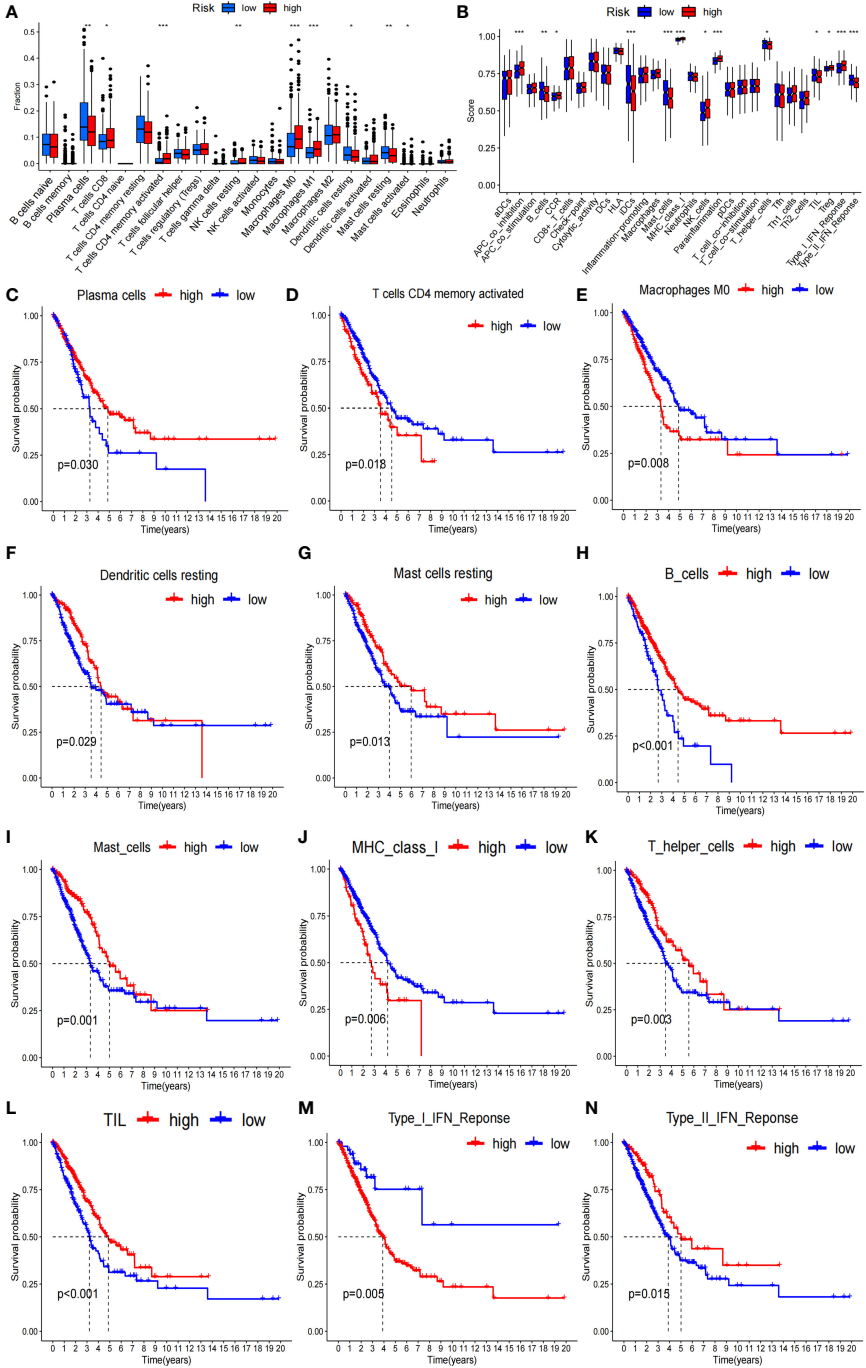
Tumor immunity analysis of the 7-g/i-DRG-DEG model. **(A, B)** Immune cell components analyses between the high- and low-risk groups by CIBERSORT analysis **(A)** and ssGSEA analysis **(B)**. **(C–G)** K−M curve between the high- and low-risk groups by CIBERSORT analysis. **(H–N)** K−M curve between the high- and low-risk groups by ssGSEA analysis. APC, Antigen-Presenting Cells; NK cells, Natural Killer cells; IFN, Interferon; DC, Dendritic Cells; TIL, Tumor Infiltrating Lymphocyte. *P<0.05; **P<0.01; *** P<0.001.

To verify whether the signature genes of the 7-g/i-DRG-DEG model are related to tumor immunity, we used the CIBERSORT and ssGSEA algorithms to compare TIICs (31). CIBERSORT analysis demonstrated that there were significant differences in plasma cells, CD8+ T cells, CD4+ memory activated T cells, resting NK cells, M0 macrophages, M1 macrophages, resting dendritic cells, and resting and activated mast cells between the high- and low-risk groups (**Figure 6A**). K−M analysis further verified that OS was related to different TIIC infiltration levels (P<0.05; **Figures 6C–G**). Similarly, ssGSEA revealed significant differences in coinhibitory effects on APCs, B cells, CCRs, iDCs, mast cells, class 1 MHC, NK cells, inflammatory cells, T helper cells, TILs, Treg T cells, the type I IFN response and the type II IFN response between the high- and low-risk groups (**Figure 6B**), as well as in OS time, which was related to different TIIC infiltration levels (**Figures 6H–N**).

**Figure 7:**
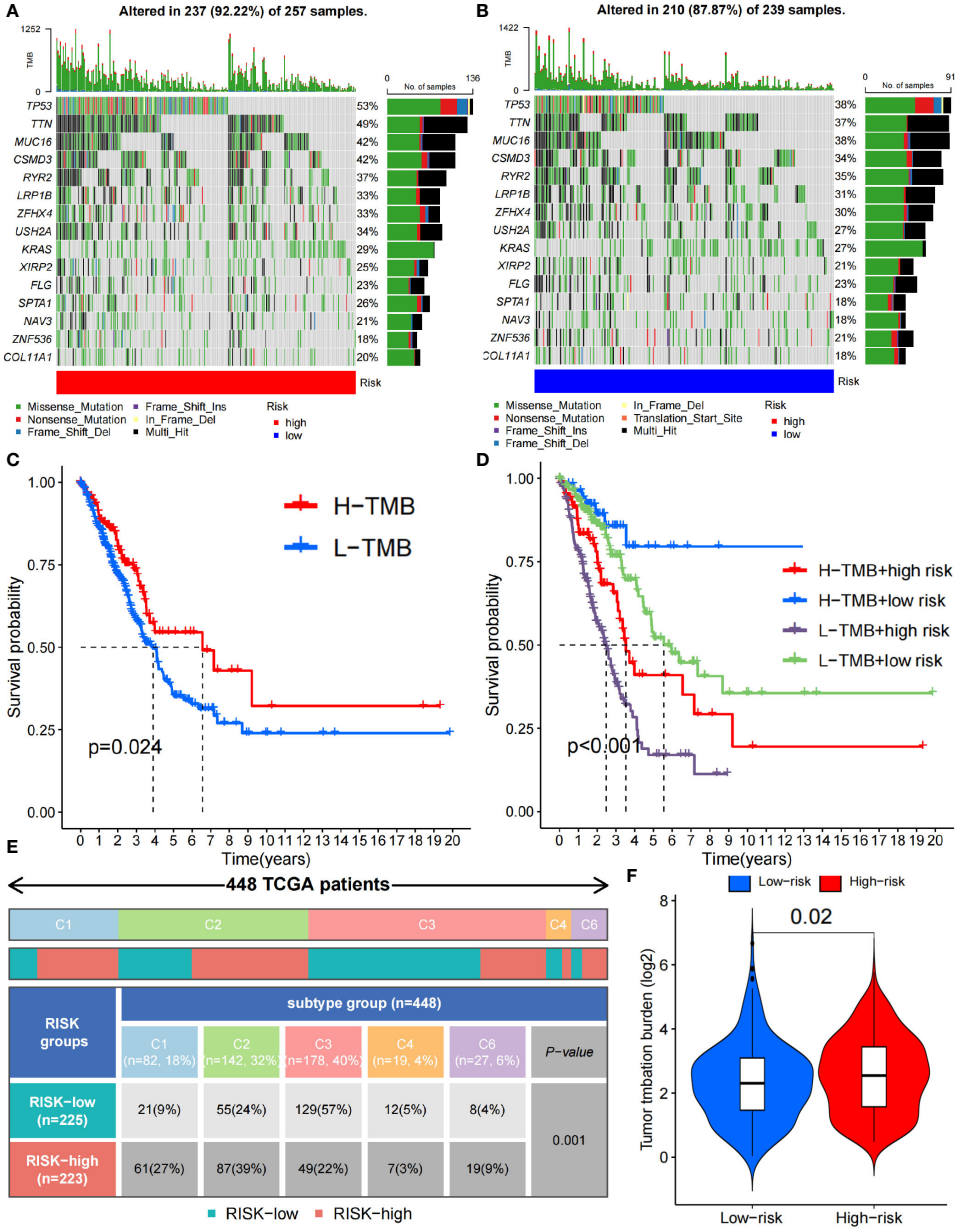
Immune characteristics between the high- and low-risk groups. **(A, B)** Waterfall plots depicting the gene mutation landscape of the 15 most frequently mutated genes in the high- **(A)** and low-risk **(B)** groups. **(C)** Survival analysis between high- and low- TMB burden. **(D)** Survival analysis among high- and low- TMB combined with high- and low-risk group. **(E)** C1-C6 immunophenotype analysis between the high- and low-risk groups (C1, wound healing; C2, IFN-γ dominant; C3, inflammatory; C4, lymphocyte depleted; C5, immunologically quiet; C6, TGF-β dominant). **(F)** TMB difference between the high- and low-risk groups. TMB, Tumor Mutational Burden.

**Figure 8:**
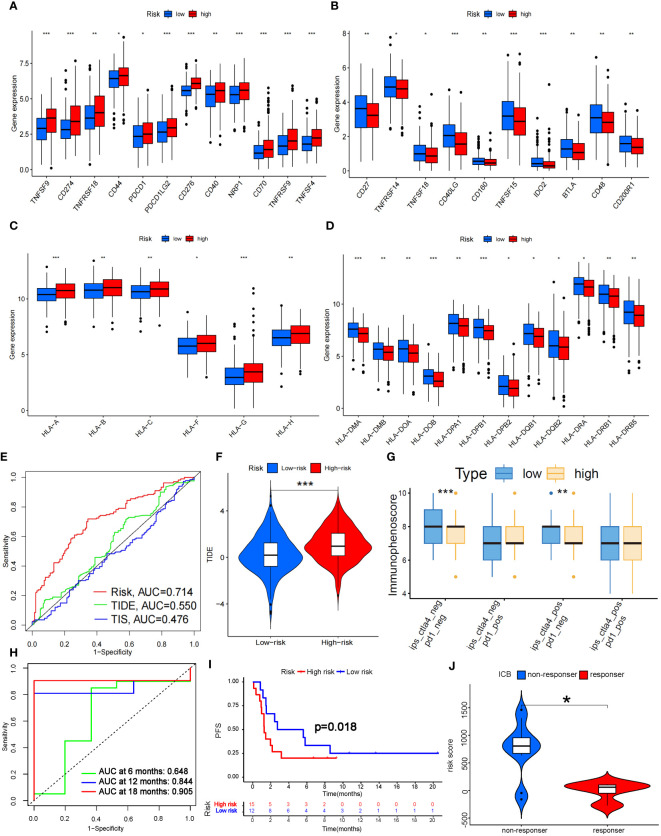
Prediction of ICB treatment efficacy. **(A-D)** Differential expression analysis of immune checkpoints **(A, B)** and human leukocyte antigens **(C, D)** between the high- and low-risk groups. **(E)** Prediction accuracy of the risk score model compared to that of the TIDE and TIS models. **(F)** TIDE scoring analysis between the high- and low-risk groups. **(G)** IPS scoring analysis between the high- and low-risk groups. **(H)** Prediction accuracy of ICB efficacy at 6, 12 and 18 months. **(I)** PFS between the high- and low-risk groups. **(J)** Response to ICB with different risk scores. HLA, Human Leukocyte Antigens; PFS, Progression Free Survival; ICB, Immune Checkpoint Blocker; TIDE, Tumor Immune Dysfunction and Exclusion; TIS, Tumor Inflammation Signature. *P<0.05; **P<0.01; *** P<0.001.

In addition, we analyzed the tumor mutation burden (TMB) in the TCGA cohort and found that, except for ZNF536 and FLG, the mutation frequency of the remaining genes with the highest mutation frequency was greater in the high-risk group (**Figures 7A, B**). In detail, the TMB of all samples in the high-risk subgroup was significantly different from that in the low-risk subgroup (P=0.02) (**Figure 7F**), and the OS time of the high-TMB subgroup was longer than that of the low-TMB subgroup (P=0.024; **Figure 7C**). The high-TMB plus low-risk subgroup had the longest OS time; in contrast, the low-TMB plus high-risk subgroup had the shortest OS time (P<0.001; **Figure 7D**). According to Thorsson’s study on dividing cancer sample cells into six immune subtypes, we also found that C1-C6 immunophenotypes were significantly different between the high-risk and low-risk groups (P=0.001) (**Figure 7E**). Furthermore, we found that there were also significant differences in the expression of immune checkpoint molecules between the high- and low-risk groups (**Figures 8A, B**). PDC1 and CD274, the most commonly used immune checkpoints, were significantly highly expressed in the high-risk group. Moreover, the HLA gene encoding MHC-I molecules was highly expressed in the high-risk group, while the genes encoding MHC-II were highly expressed in the low-risk group (**Figures 8C, D**).

Fortunately, the predictive accuracy of our risk-scoring model was comparable to that of the TIDE and TIS models (**Figure 8E**). We further verified that the high-risk group in our model had a significantly greater score than the low-risk group by TIDE scoring (P<0.001; **Figure 8F**), which indicated that the high-risk patients suffered a worse response to immune checkpoint inhibitor (ICI) treatment. However, there was no significant difference in the PD-1-positive group according to the Immunephenoscore (IPS), which indicated that it was difficult for our model to predict the efficacy of PD-1 blockers. The low-risk group with CTLA-4 positive had higher scores than did the high-risk group (P<0.01; **Figure 8G**), which indicated that the low-risk group might possess stronger CTLA-4 blocker sensitivity. CTLA4 is upregulated in activated regulatory T cells (Tregs) and can bind to CD80 or CD86 on the surface of antigen-presenting cells, thus “shutting down” tumor immunity (32). We noticed that Treg numbers decreased significantly in the high-risk group according to ssGSEA, which further confirmed the predictive effect of our model on CTLA-4 immune checkpoints. To further verify the predictive effect of this risk model on immunotherapy efficacy, we selected data sets (GSE135222 and GSE126044) which contained lung cancer samples after treatment with Ipilimumab, Nivolumab or Pembrolizumab (33). As a result, we found this model had a good predictive effect on immune efficacy, with AUC at 12 and 18 months both exceeding 0.8 (**Figure 8H**). The PFS of the low-risk group was significantly longer than the high-risk group (P<0.05; **Figure 8I**). In addition, as the risk score increased, the number of non-responders to ICB treatment was also significantly more than that of responders (P<0.05; **Figure 8J**). These verifications further illustrated that the 7-g/i-DRG-DEG model could predict the ICB efficacy in NSCLC accurately.

## Discussion

From the discovery of aerobic glycolysis to the pentose phosphate pathway (PPP), characterizing the special metabolism of tumor cells has always been a research direction for breakthrough tumor treatment (34). When the oxygen supply cannot meet the energy production needs of mitochondria, tumor cells increase glycolysis to fill the energy gap caused by insufficient ATP, thereby preventing tumor cell death caused by hypoxia (35). Tumor cells steal more glucose by overexpressing glucose transporter proteins, and excess raw material increases glycolysis levels in tumor cells (36). Simply put, tumor cells reprogram the normal process of glucose metabolism to gain an advantage in confronting immune cells and competing with normal tissue cells (37). To further confirm this conclusion, we selected a cohort of 600 samples from the TCGA database and performed differential gene analysis on glucose metabolism-associated genes. We found that genes related to hypoxia, glycolysis, glucose transport, and the PPP were differentially upregulated, which indirectly explained why the hypoxic environment of tumor cells led to a high consumption and low production state in the glucose metabolism pathway. Therefore, the highly expressed glucose transporter (GLUT) plays an important role in maintaining this state of tumor cells.

Nevertheless, an important reason for the occurrence of disulfidptosis is the insufficient supply of reduced NADPH during glucose starvation, which leads to the abnormal accumulation of cystine and other disulfides in cells with high SLC7A11 expression. As a new form of PCD, disulfidptosis of tumor cells can be further exacerbated by limiting glucose uptake via the use of GLUT inhibitors (38). We further performed differential expression analysis of 22 DRGs, and the results showed that SLC7A11, a key gene involved in disulfidoptosis, was differentially elevated in tumors. Moreover, we found that DRGs expression was also differentially increased in several important TIICs involved in tumor suppressive immunity, such as CD4+ T cells and tumor-associated macrophages (TAMs). Therefore, we infer that GLUT inhibitors can also have therapeutic effects on the suppressive tumor immune microenvironment (39).

Immunotherapy plays an irreplaceable role in tumor treatment. However, ICB has great limitations in clinical application. On the one hand, only a few tumors highly express immune checkpoints; on the other hand, the efficacy of ICB is uncertain even in some immune checkpoint-positive tumors (40). Moreover, in some EGFR-mutated tumors, ICB may even lead to tumor hyperprogression (41). There is an urgent need for effective means to predict immune efficacy in the clinic. Therefore, we constructed models related to immunity and glucose metabolism to address this issue.

We extracted a total of 3337 genes through the GSEA, IMMORT and INNATE datasets. We selected 905 genes that were differentially expressed in tumors (g/i-DEG) and 707 genes related to disulfidptosis (g/i-DRG) and then intersected the two gene sets to obtain 128 differentially expressed disulfidptosis-related genes (g/i-DRG-DEG). Thirty-seven “risk” genes were identified by Cox regression analysis of OS time. Finally, through LASSO Cox regression analysis, we constructed a 7-g/i-DRG-DEG model containing 7 signature genes (ARRB1, LIFR, PDGFB, LGR4, KIF20A, NT5E and PHKA1) in the training cohort. We verified the reliability of the model in the validation set and 3 external GEO datasets stratified by different risk score groups. The results proved that this model can better predict the survival of tumor patients than can the classical TNM staging and risk score models.

We studied the relationship between the 7-g/i-DRG-DEG model risk score and cellular functions. Through differential gene analysis, we found that the NT5E and KIF20A genes, which are highly related to tumor development, were differentially elevated in the high-risk group. The GSEA, KEGG and GO analyses revealed enrichment of genes involved in various functions and pathways, including the cell cycle, cytoskeleton movement activity, microtubule movement activity, organelle division, nuclear division, chromosome segregation, chromosome-related regions, organelles involved in cell division and extracellular matrix containing collagen. Notably, the genes in the high-risk group were positively correlated with the cell cycle, ECM receptor interaction, regulation of the actin cytoskeleton, and regulation of mitosis, indicating that the genes in the high-risk group had stronger cell proliferation and division functions. This result is consistent with the clinical observation that poorly differentiated and highly proliferative tumor cells usually have a worse prognosis (42). As mentioned before, disulfidptosis relies on abnormal cross-linking of disulfide bonds between cytoskeletal proteins, and the high-risk group also exhibited a greater ability to regulate the actin cytoskeleton (43).

Given that the immune response of the tumor microenvironment is an important factor in determining tumor cell aggressiveness and progression (44), we further verified the immune impact of the 7-g/i-DRG-DEG model. By comparing TIICs through the CIBERSORT and ssGSEA algorithms respectively, we found that immune cells with direct tumor killing functions, such as plasma cells and CD8+ T cells, were significantly reduced in the high-risk group, while immune cells with auxiliary functions, such as M0 macrophages, M1 macrophages, and activated CD4+ T cells, were significantly increased in the high-risk group. However, tumor-associated macrophages (TAMs) have been a hot topic of cancer research in recent years. An increasing number of studies have proven that TAM infiltration is strongly correlated with the poor prognosis of tumor patients due to a series of functions that promote tumor development, such as supporting tumor cell proliferation, invasion, and metastasis (45). We also noted that in another similar disulfidptosis-related model, the expression of signature genes was positively correlated with M1 cell migration and invasion, indicating that there is obvious tropism of M1 cells in the high-risk group (46).

In addition, CD8+ T cells in the high-risk group were more susceptible to immune checkpoint effects. We found that the immune checkpoint molecule CTLA-4, which is currently widely used in clinical applications, was significantly overexpressed in the high-risk group, indicating that the high-risk group may have a suppressive TME that is more unfavorable for tumor immunity. Subsequently, the accuracy of the prediction of ICB efficacy was verified to further illustrate that this model has reference value for the clinical application of ICB. Specifically, The high expression of HLA-I class molecules in the high-risk group also confirmed this conclusion. HLA-I class molecules stimulate cytotoxic immune responses by binding CD8+ T cells, and HLA-II class molecules bind to CD4+ T cells (47). An increase in HLA class I molecules in the high-risk group indicated a decrease in CD8+ T cells. HLA-II class molecules were lower in the high-risk group, further indicating that CD4^+^ immune cells increased, which proved by immune infiltration analysis. For the immune phenotype, the expression of angiogenic genes was enhanced in the high-risk group, which had a high proliferation rate, and Th2 cells were prone to acquired immune osmosis, which stimulated the proliferation of CD4+ T cells and B cells. These results indicate that the TME in the high-risk group was more inclined to exhibit a “cold immune” phenotype, which is associated with greater tumor malignancy and a more tolerant immune environment (48).

In summary, benefiting from the development of bioinformatics technology, we constructed a 7-gene signature prediction model based on TCGA LUAD patient data and evaluated patient tumor prognosis risk through signature gene expression. We demonstrated the reliability of this model and further validated its value in predicting tumor immunity through immune infiltration analysis. Such studies will help to develop more personalized treatment strategies in the future, promote the development of new drugs, and ultimately extend the survival expectancy of cancer patients.

## Data availability statement

The datasets presented in this study can be found in online repositories. The names of the repository/repositories and accession number(s) can be found in the article/**Supplementary Material**.

## Ethics statement

The studies involving humans were approved by 920th Hospital of Joint Logistics Support Force. The studies were conducted in accordance with the local legislation and institutional requirements. Written informed consent for participation was not required from the participants or the participants’ legal guardians/next of kin in accordance with the national legislation and institutional requirements.

## Author contributions

KZ: Conceptualization, Writing – original draft. GL: Validation, Visualization, Writing – original draft. QW: Data curation, Formal analysis, Writing – original draft. XL: Conceptualization, Writing – original draft. HC: Resources, Writing – review & editing. FL: Methodology, Project administration, Writing – review & editing. SL: Validation, Visualization, Writing – review & editing. XS: Resources, Supervision, Writing – review & editing. YL: Funding acquisition, Resources, Supervision, Writing – review & editing.
