# Supplementary figures and images for: A disulfidptosis-related glucose metabolism and immune response prognostic model revealing the immune microenvironment in lung adenocarcinoma
Source: Front Immunol. 2024 Jul 18;15:1398802. doi: 10.3389/fimmu.2024.1398802 (PMC11291233; doi:10.3389/fimmu.2024.1398802)

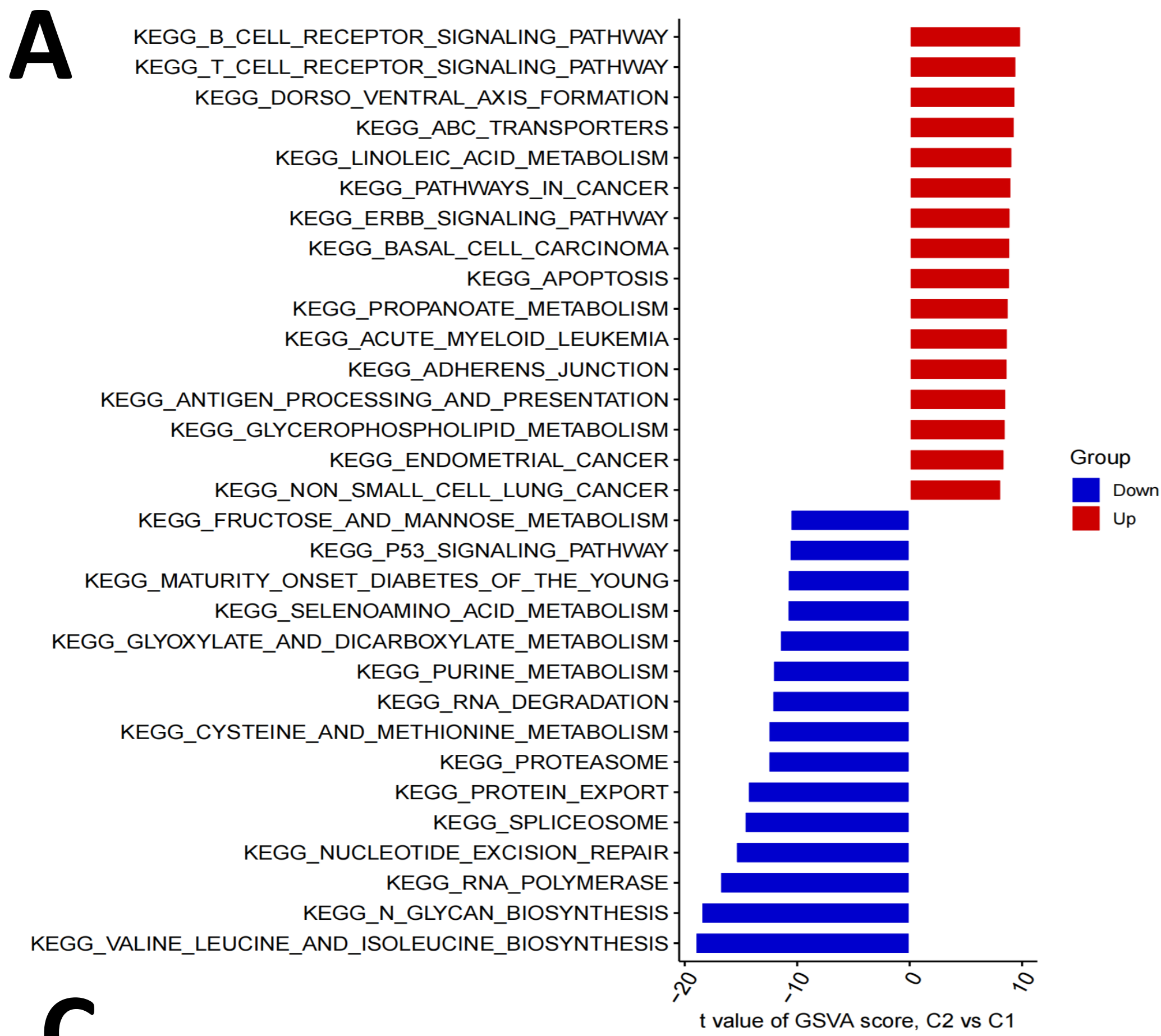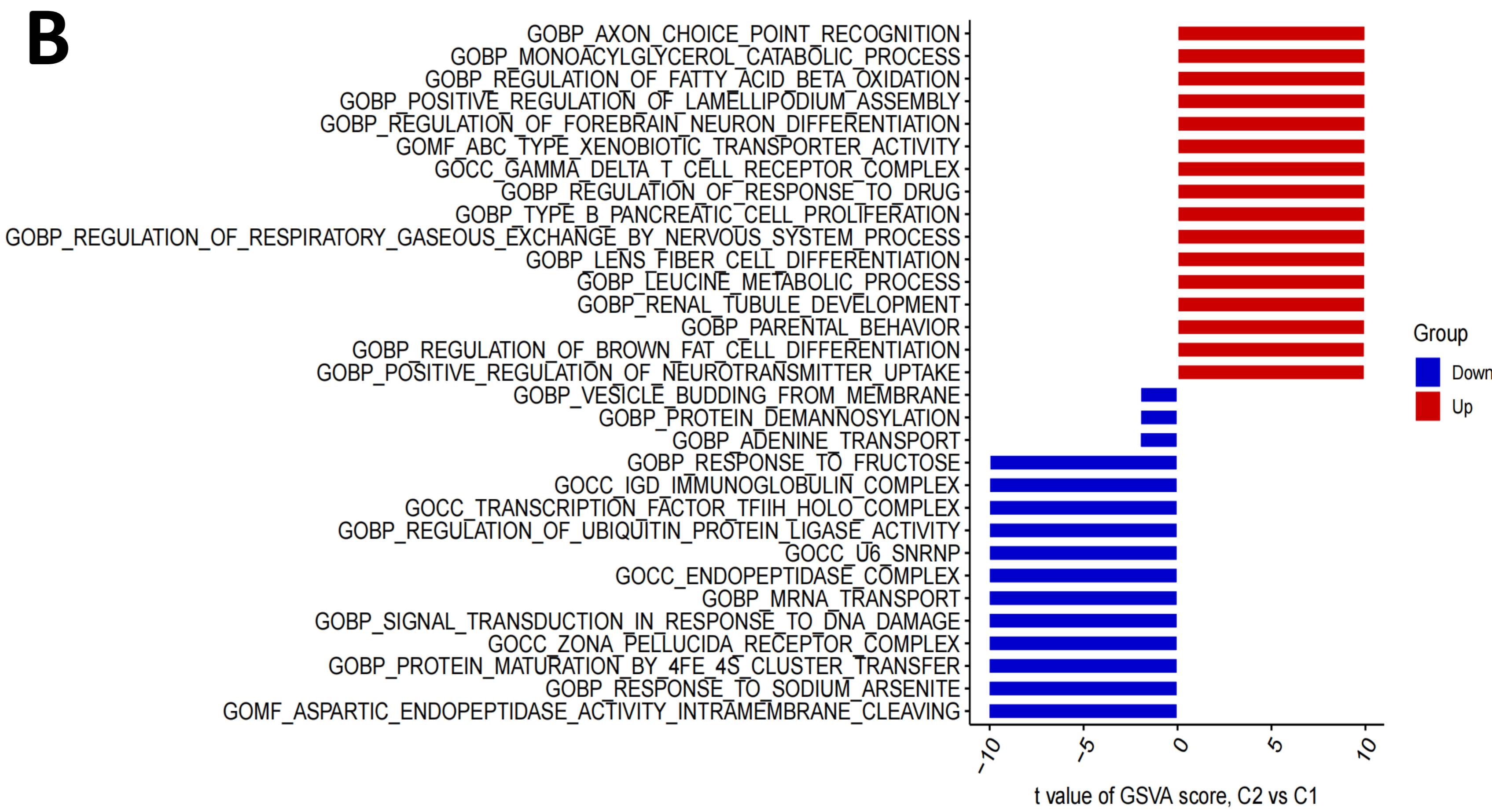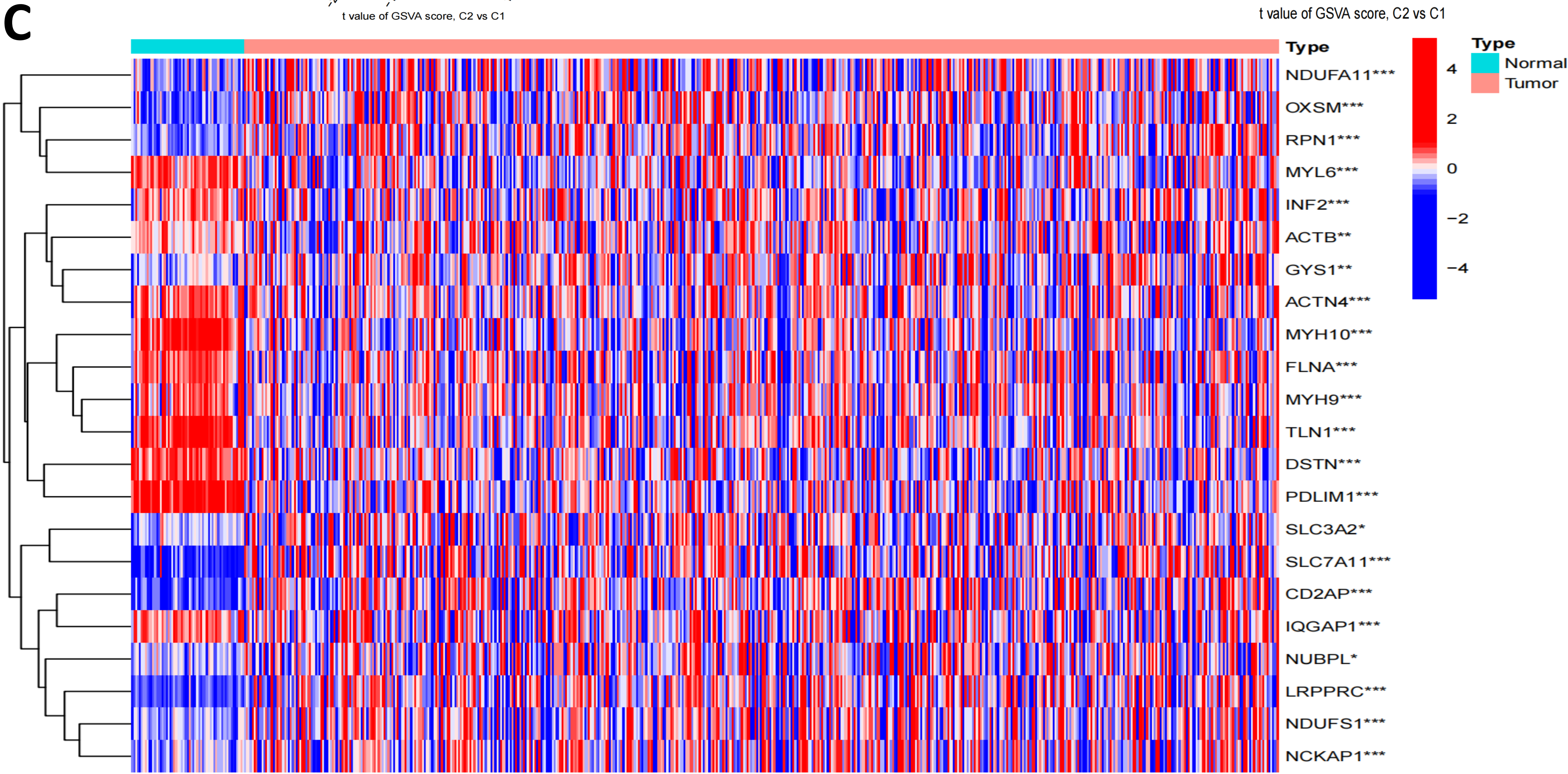

Supplement: Supplementary file 1 [file Image_1.pdf]

GSE26939 cohort

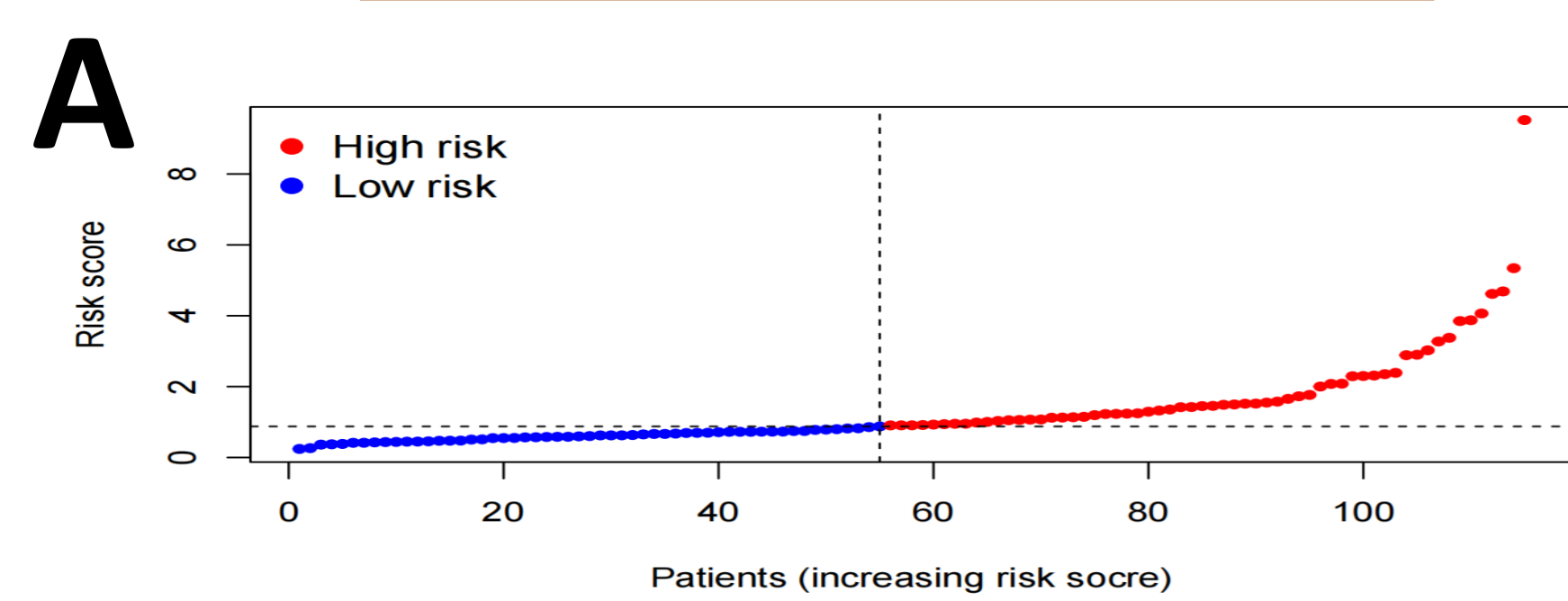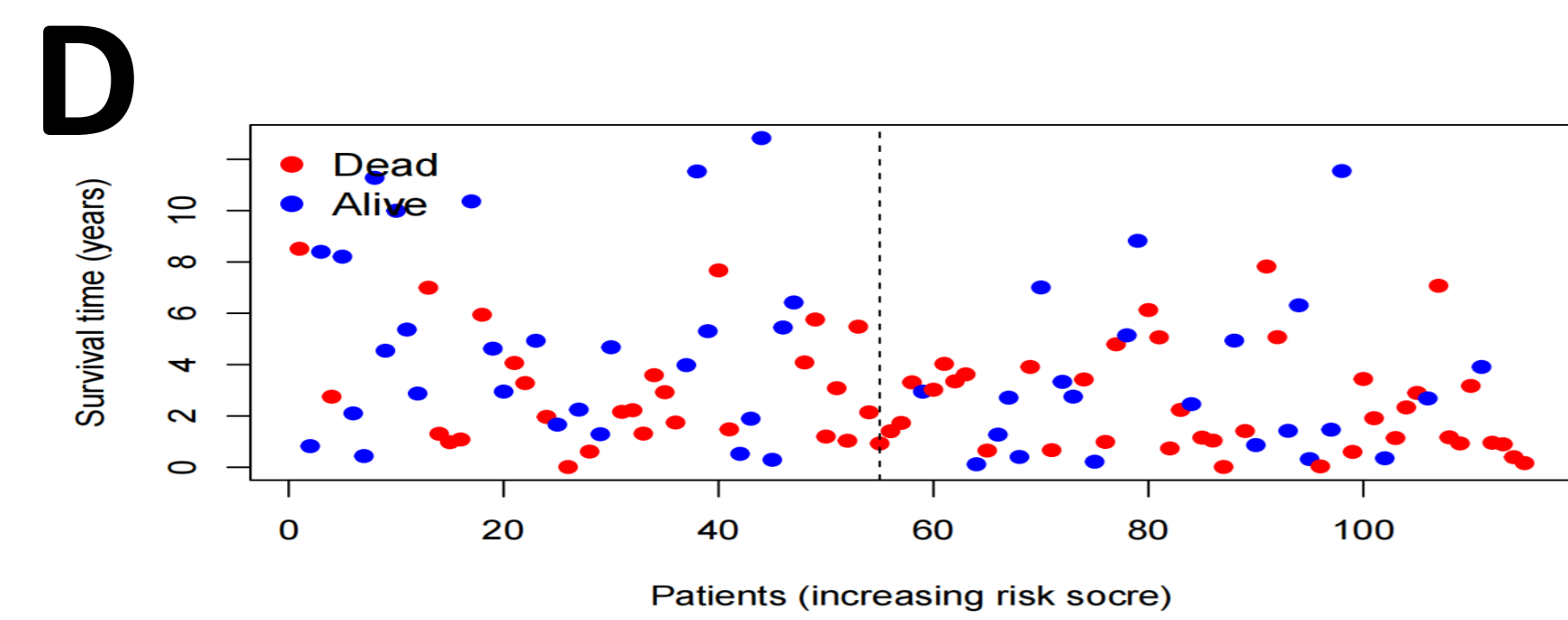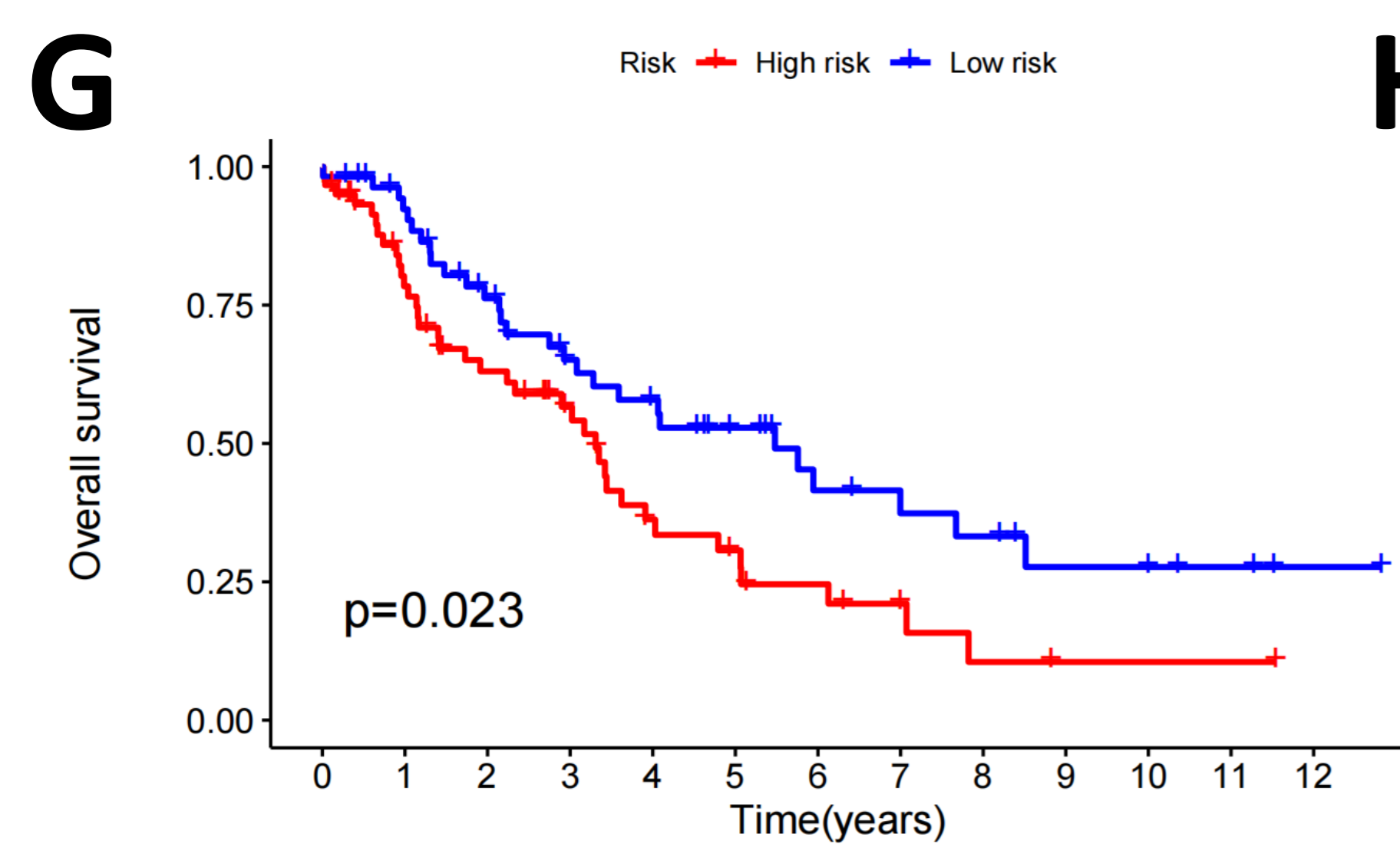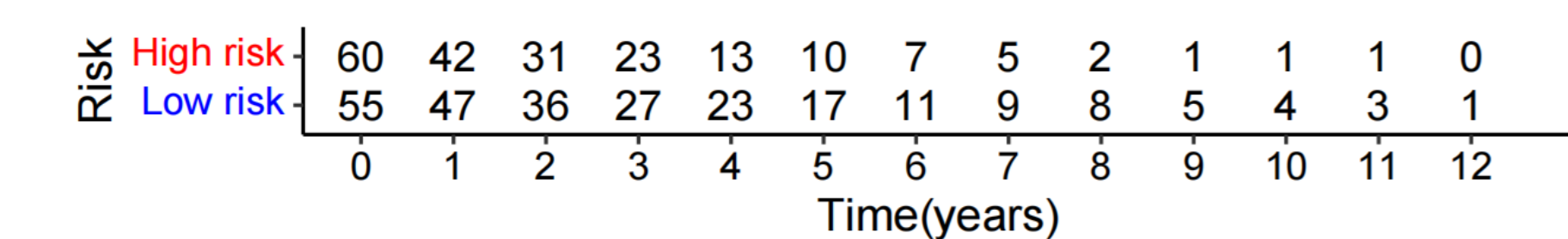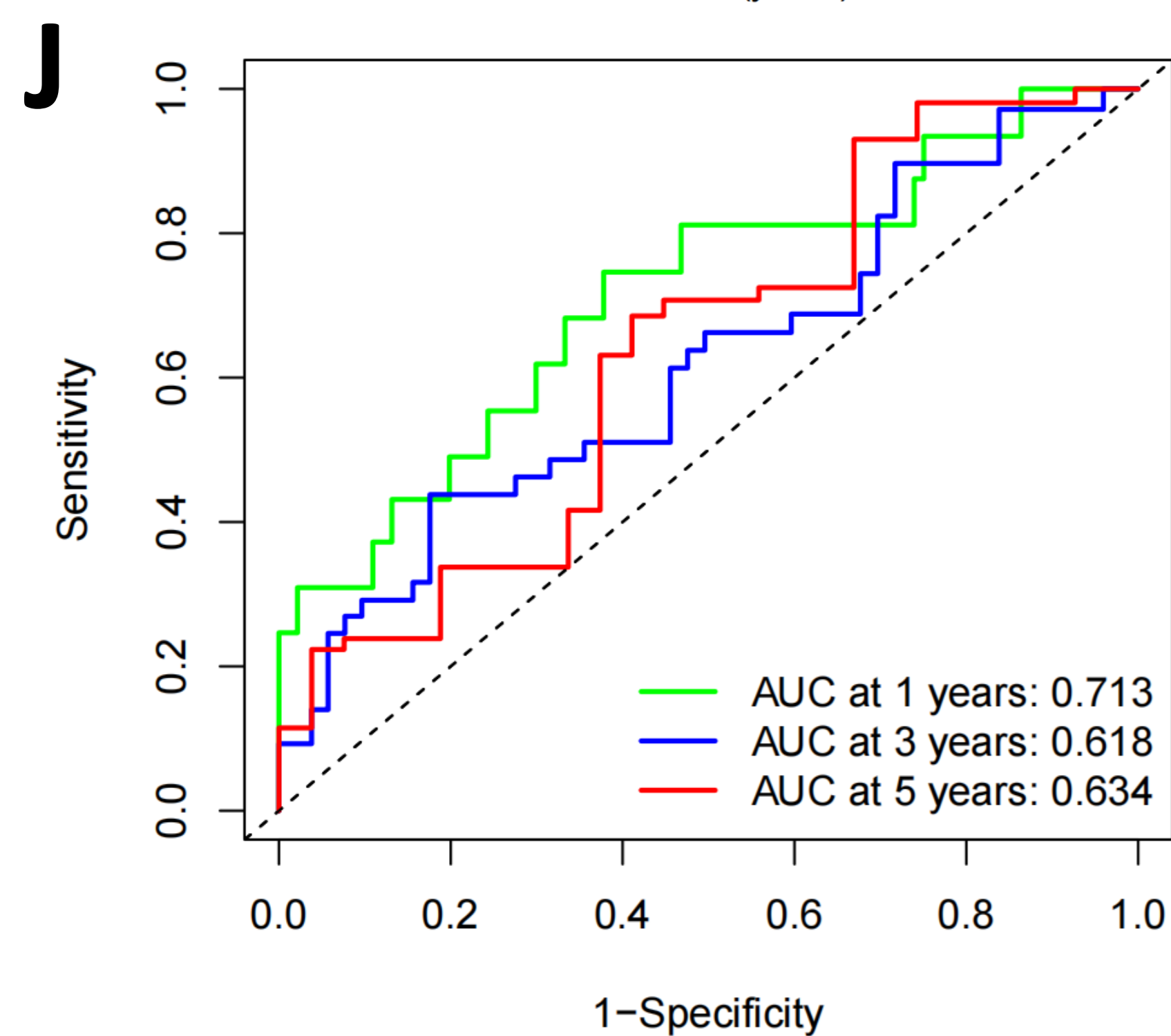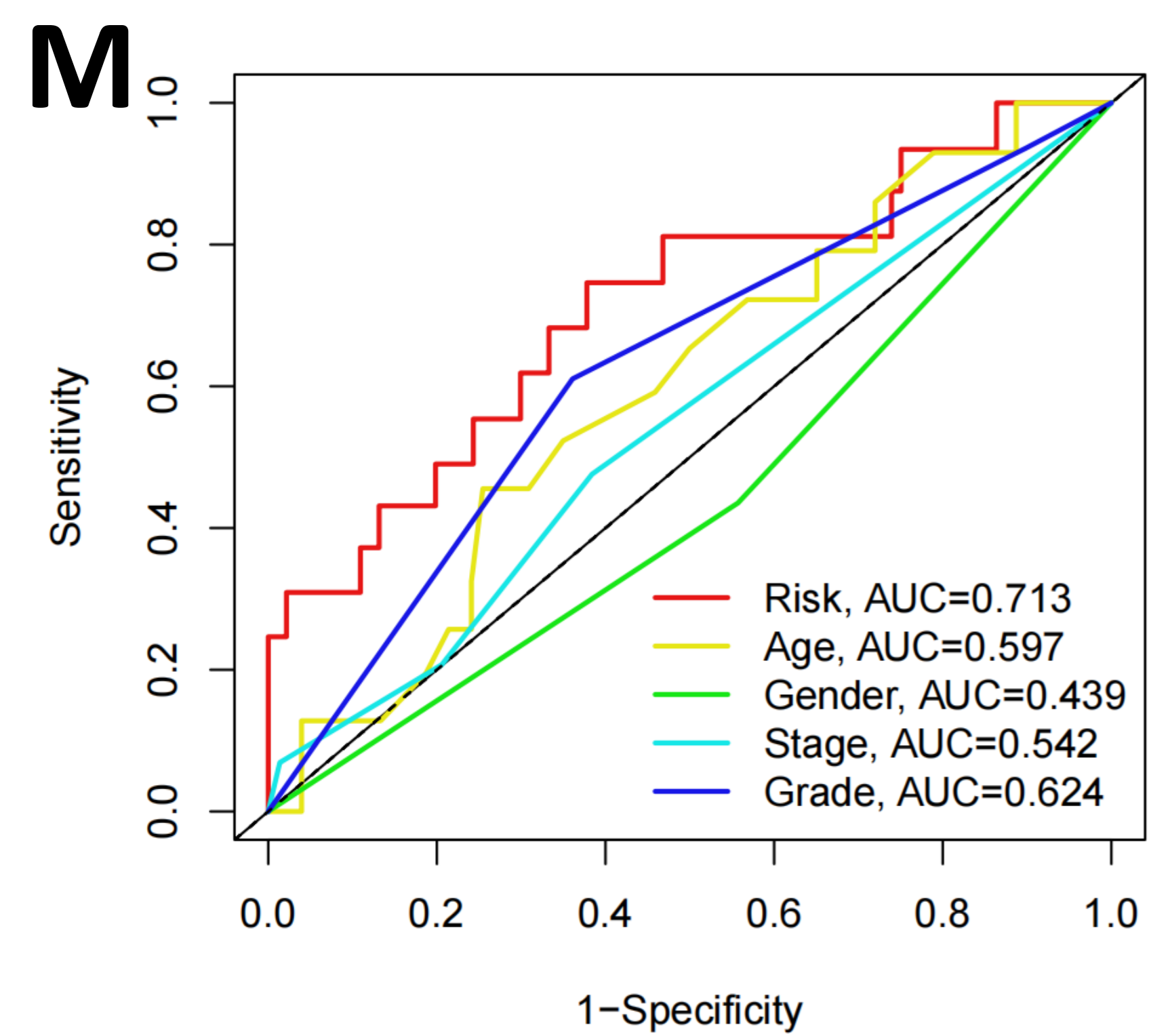

GSE68465 cohort

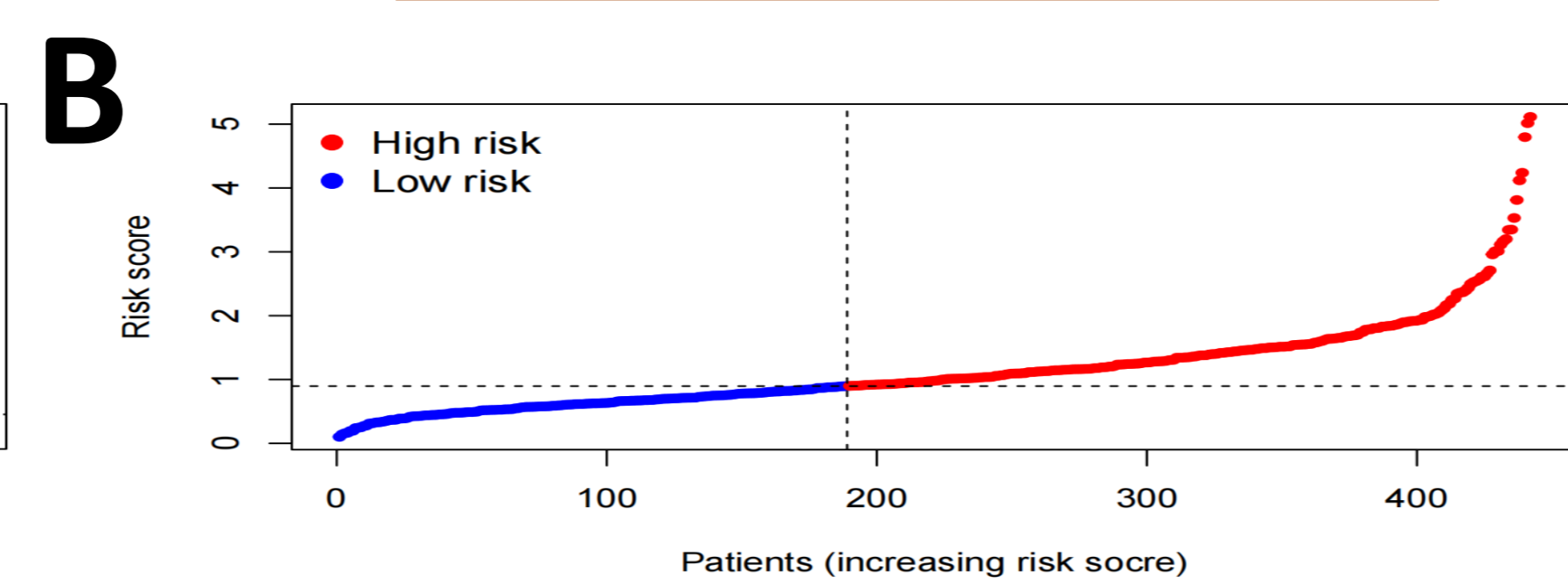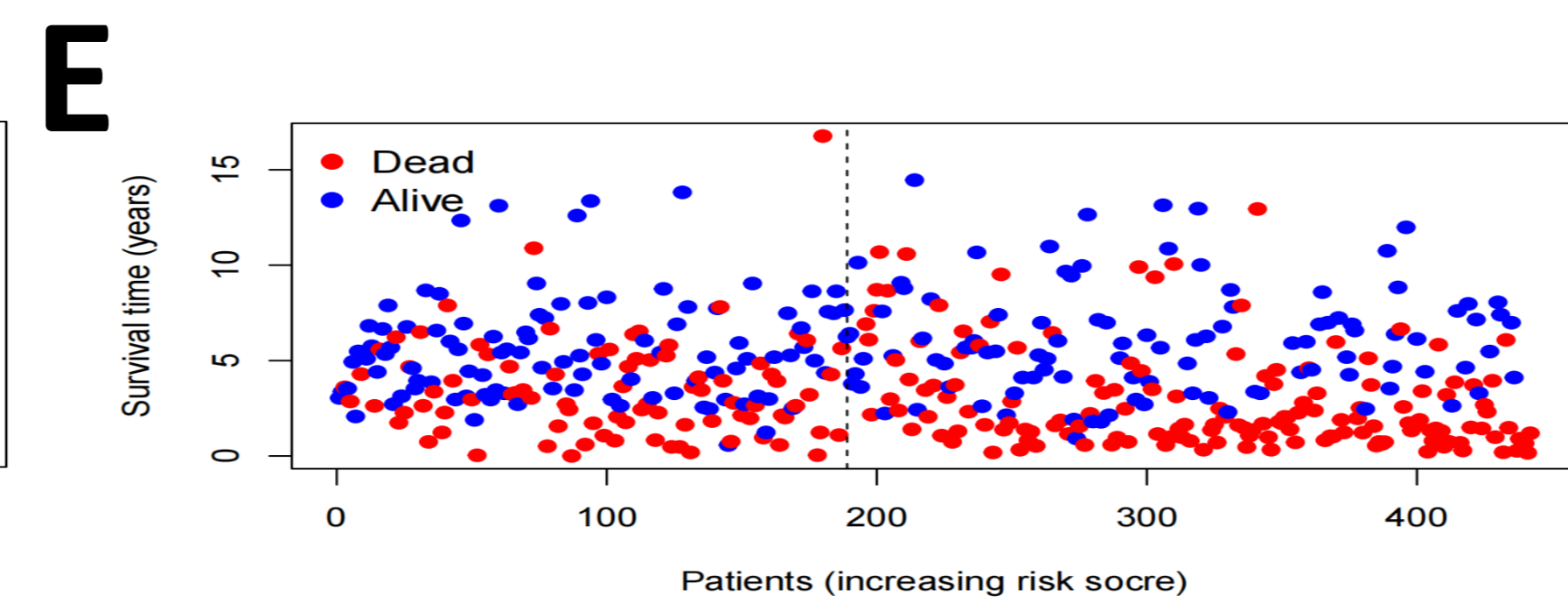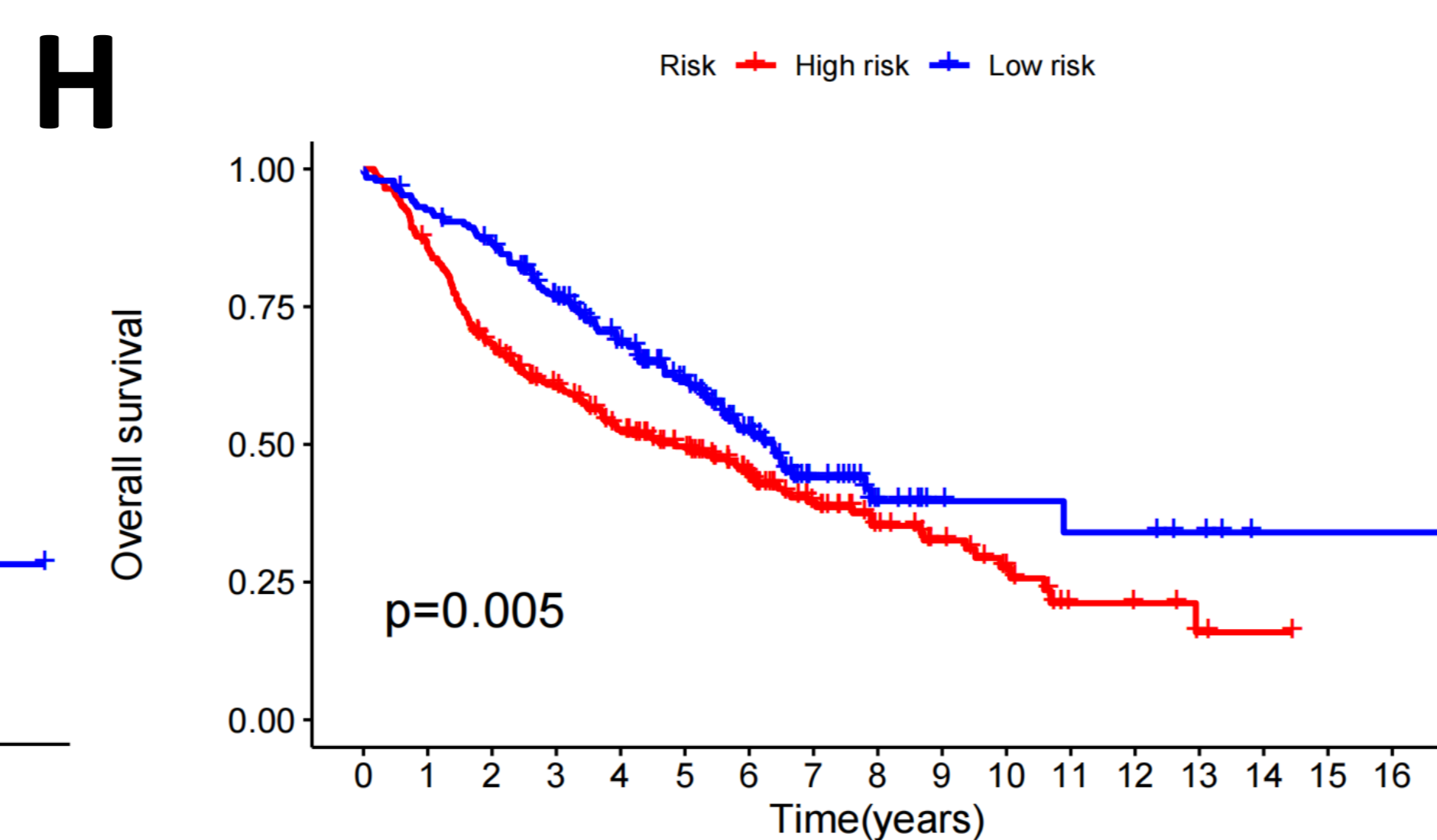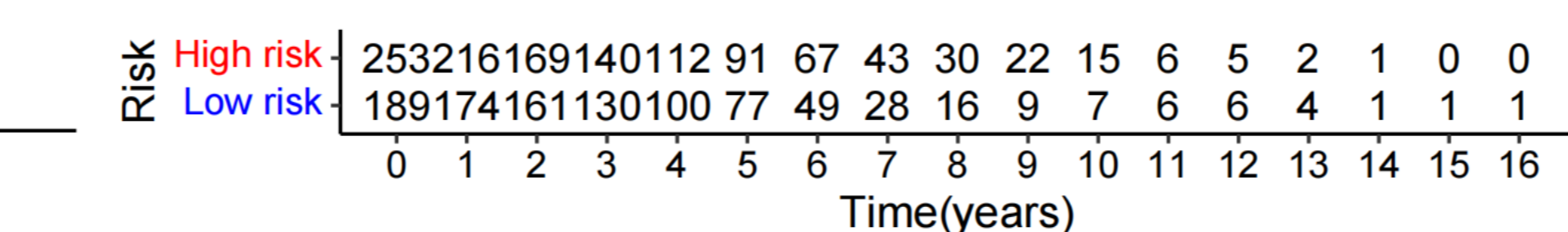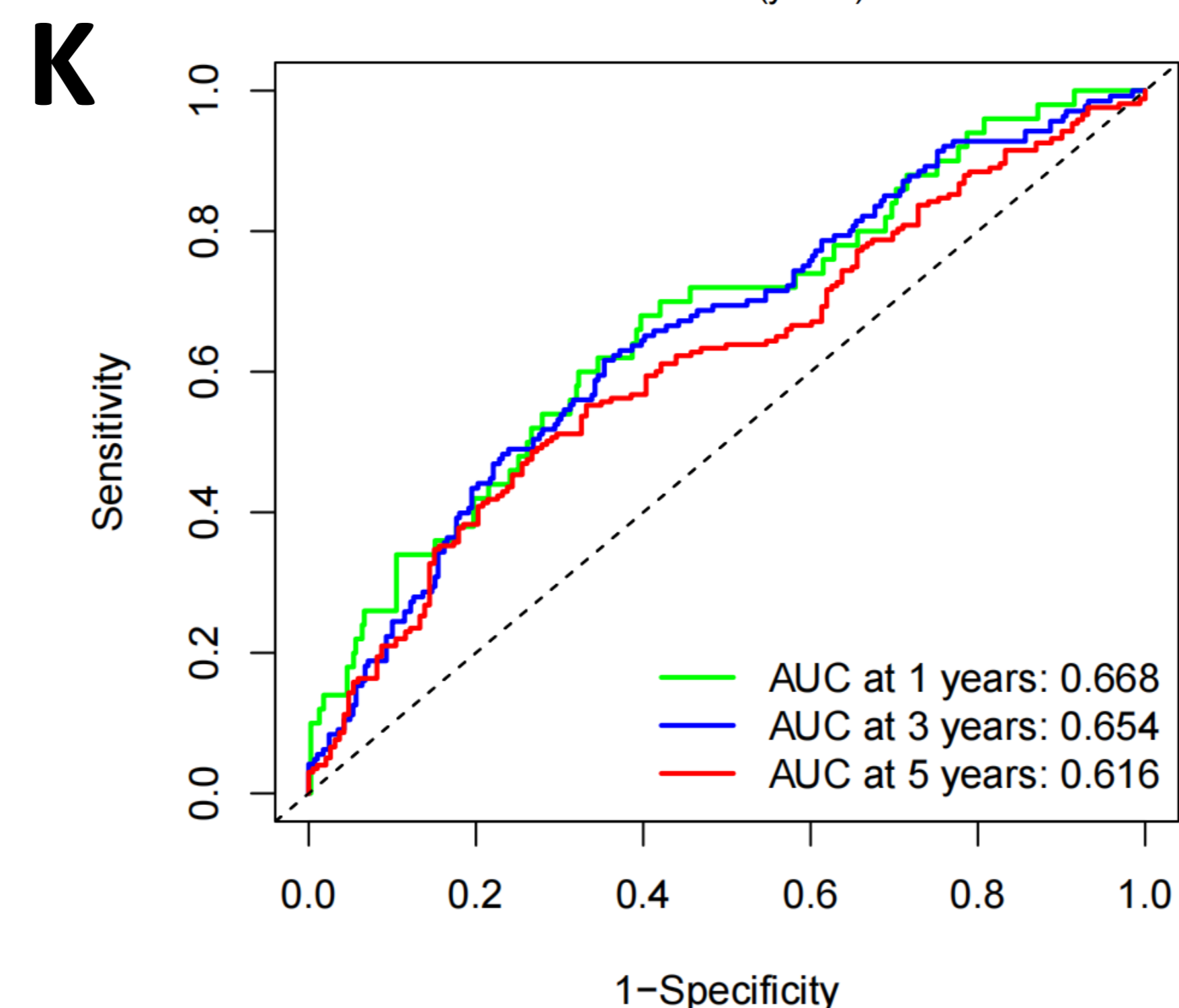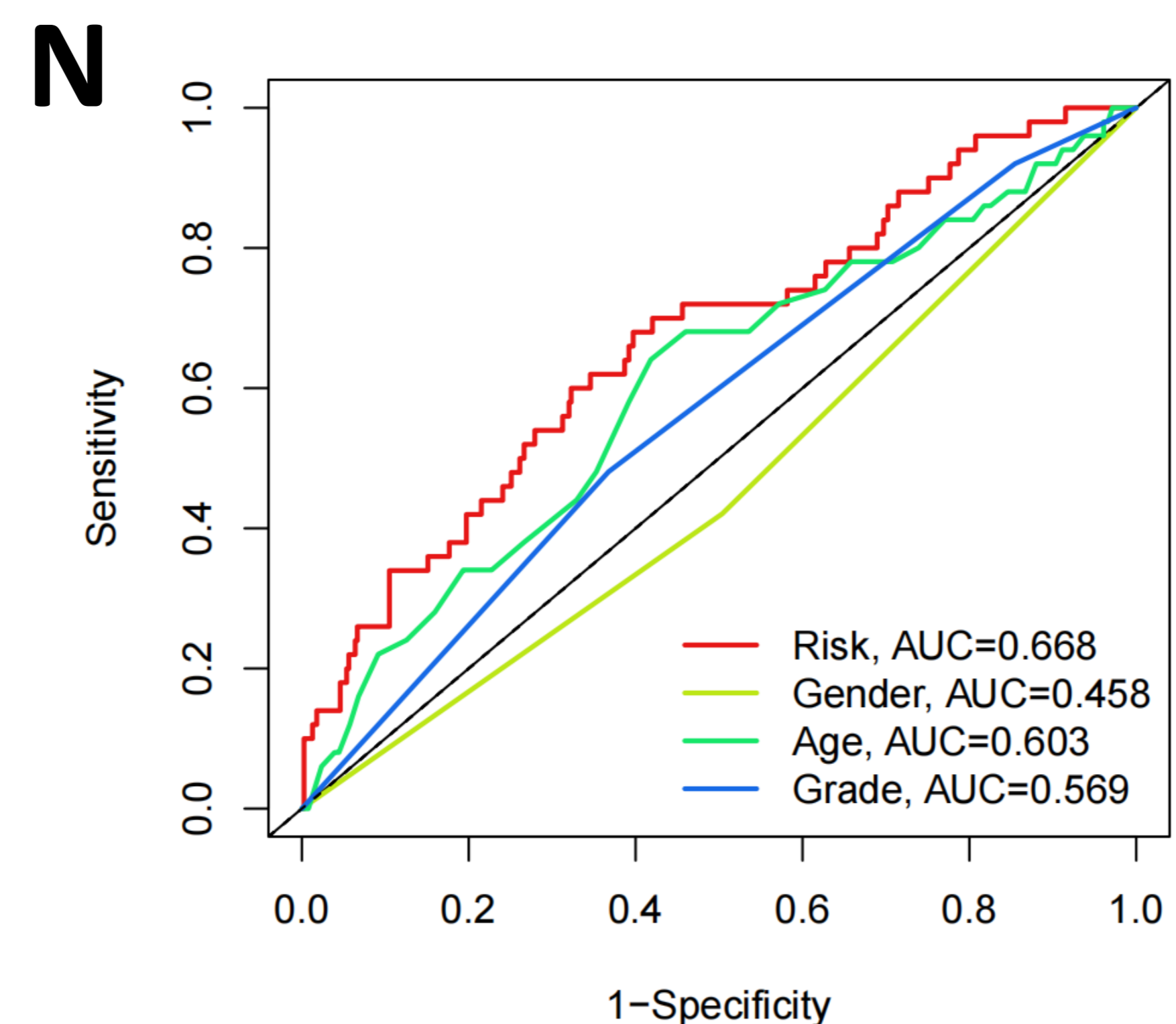

GSE72094 cohort

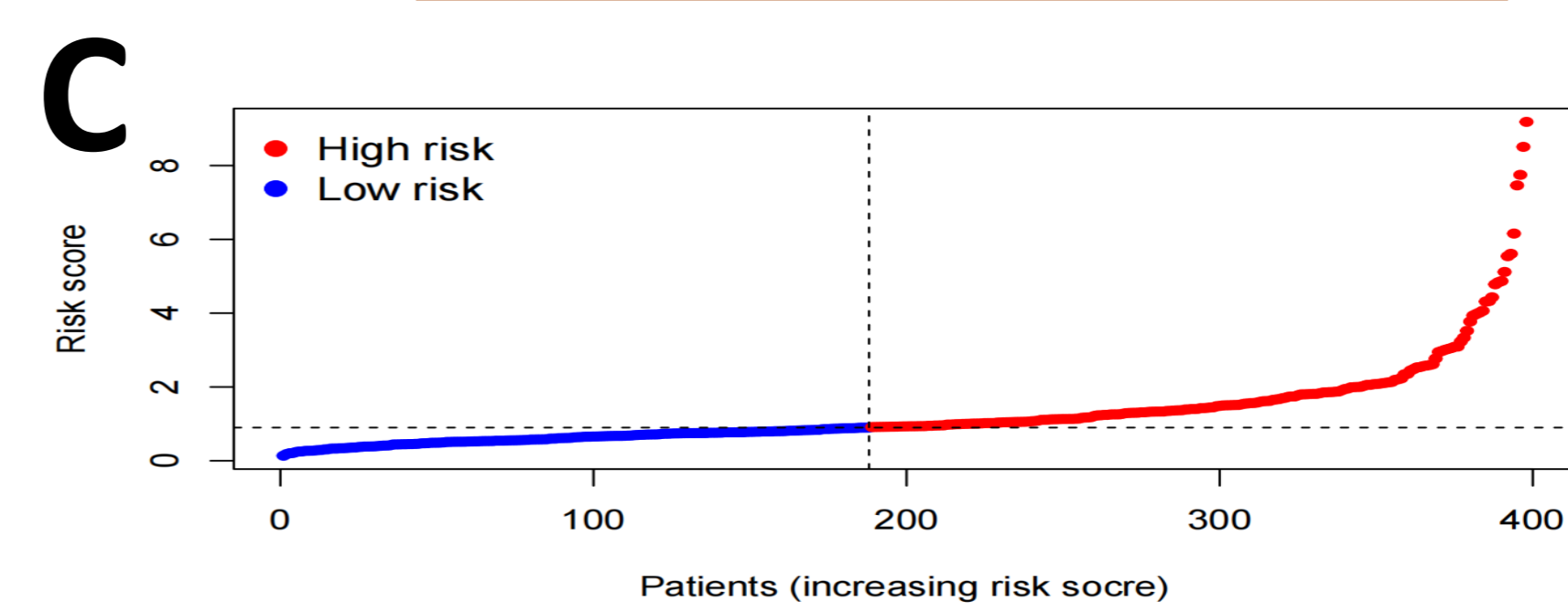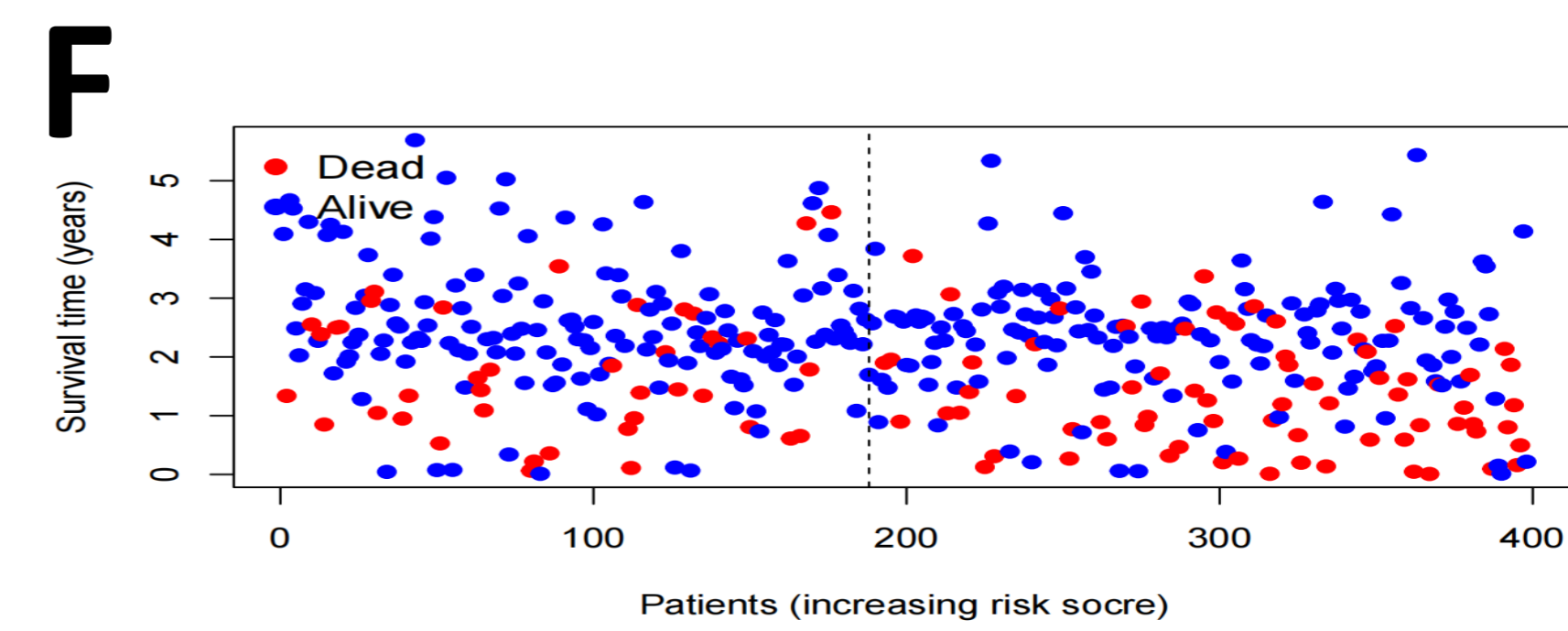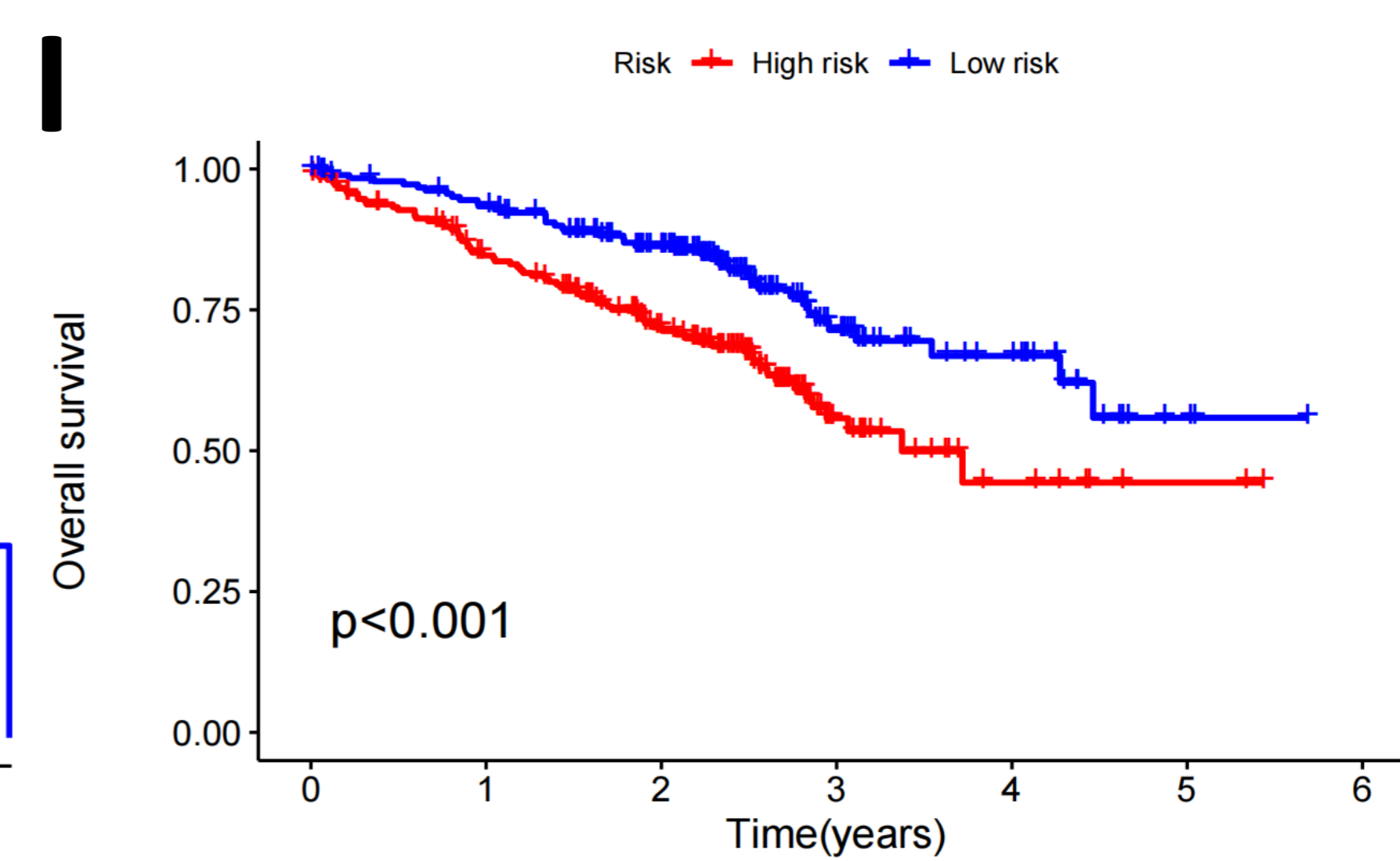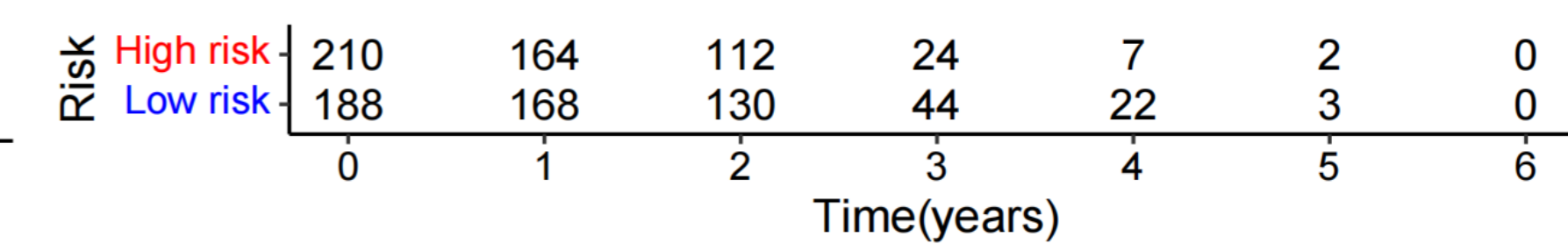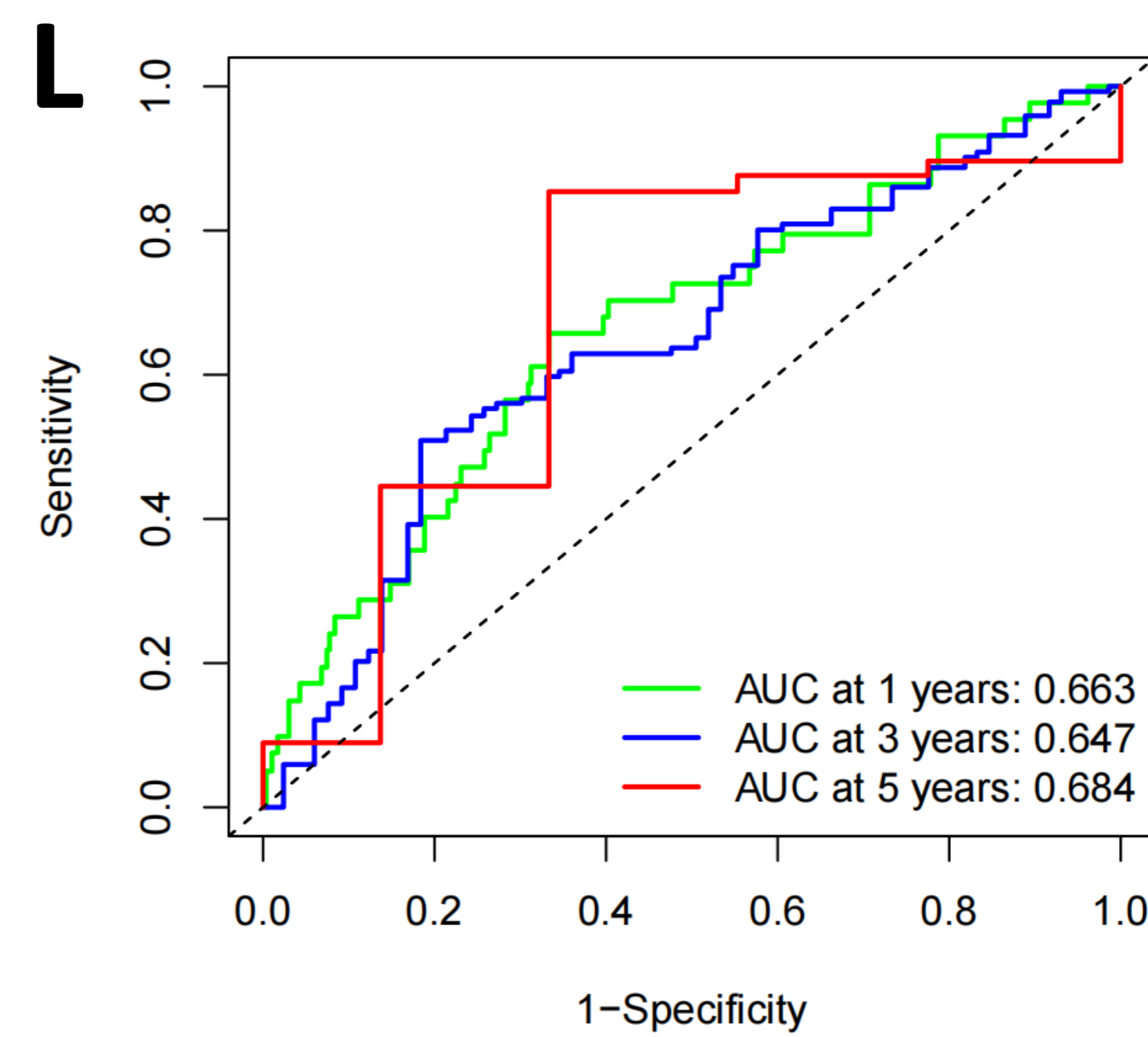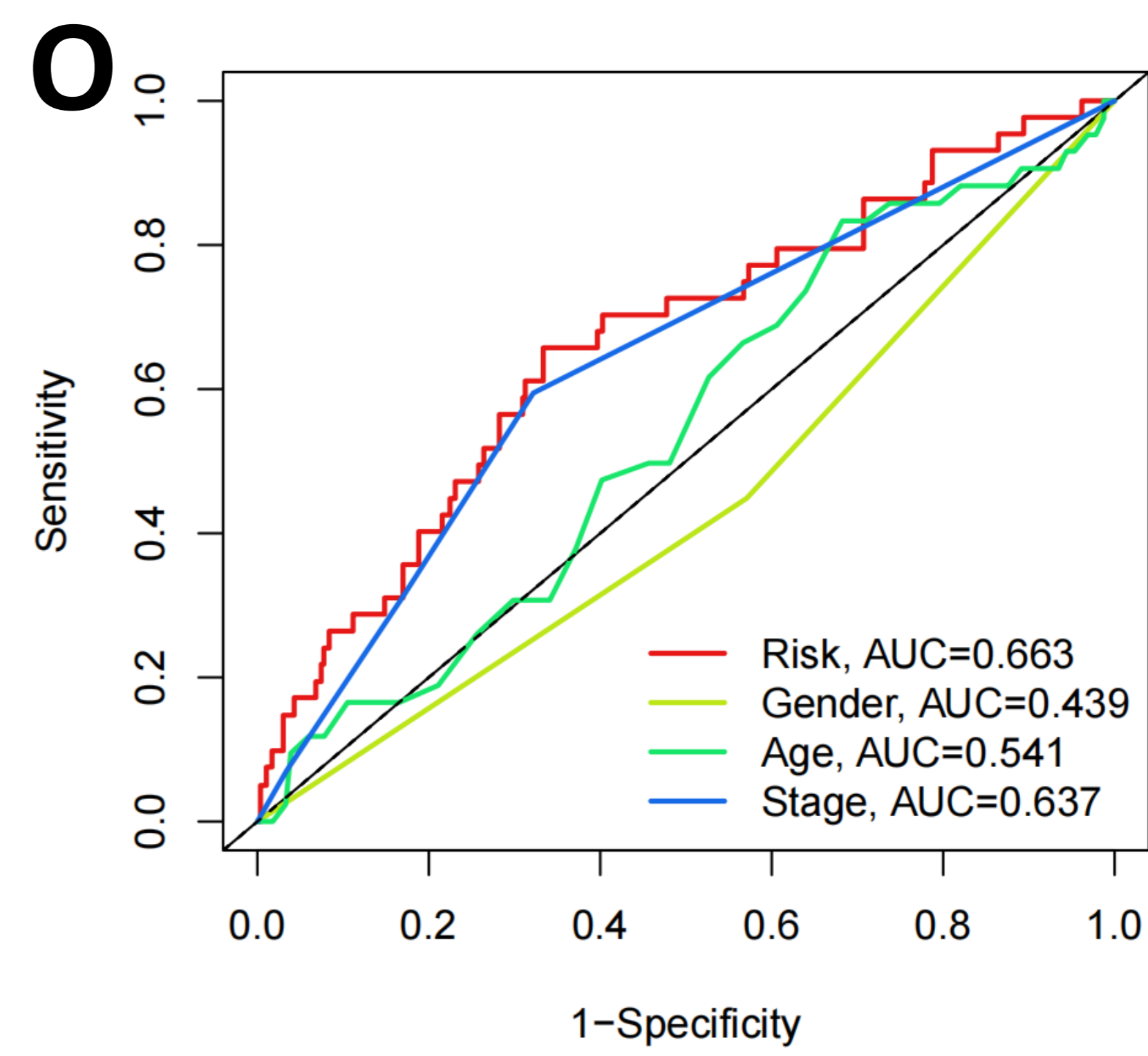

Supplement: Supplementary file 2 [file Image_2.pdf]

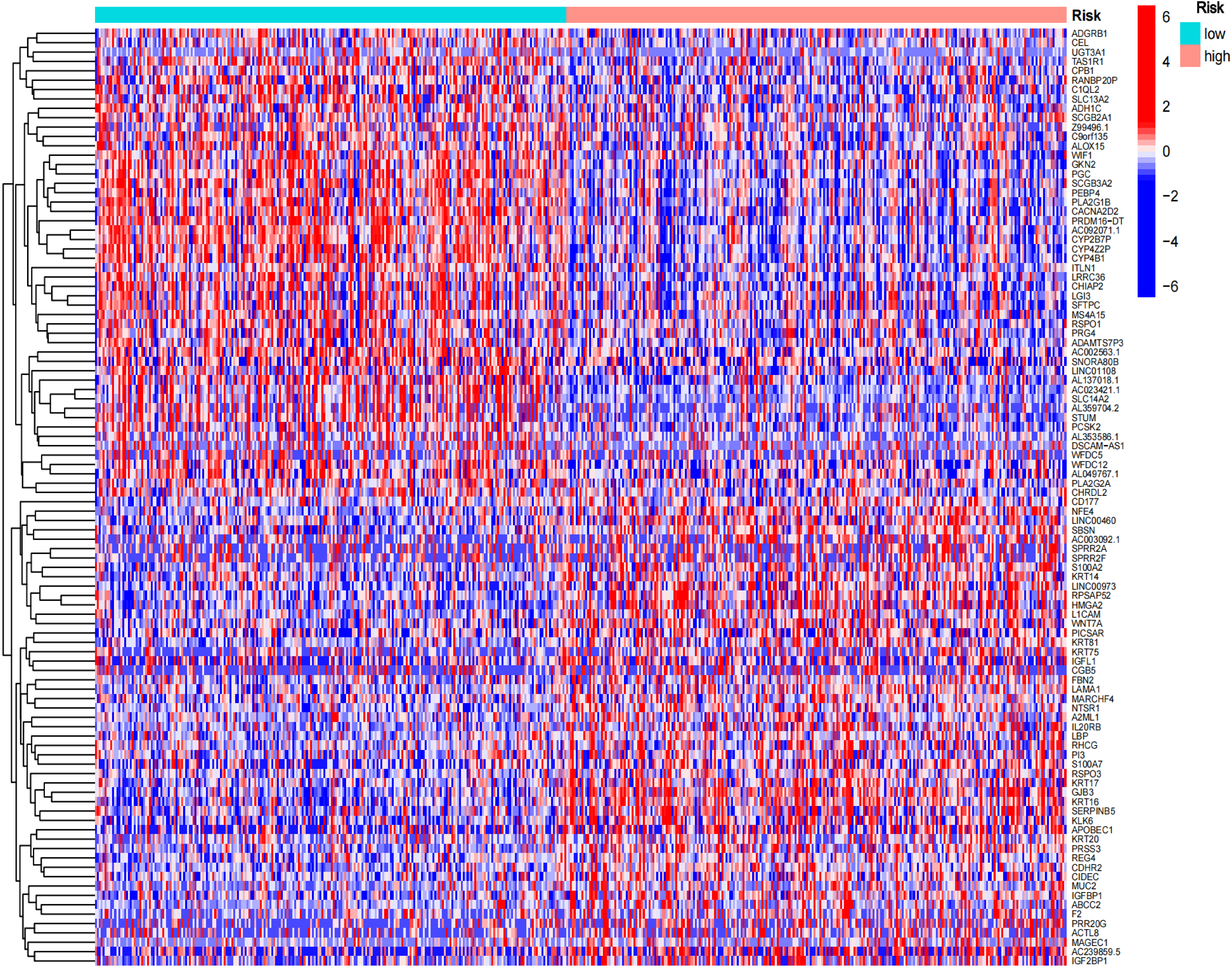

Supplement: Supplementary file 3 [file Image_3.pdf]

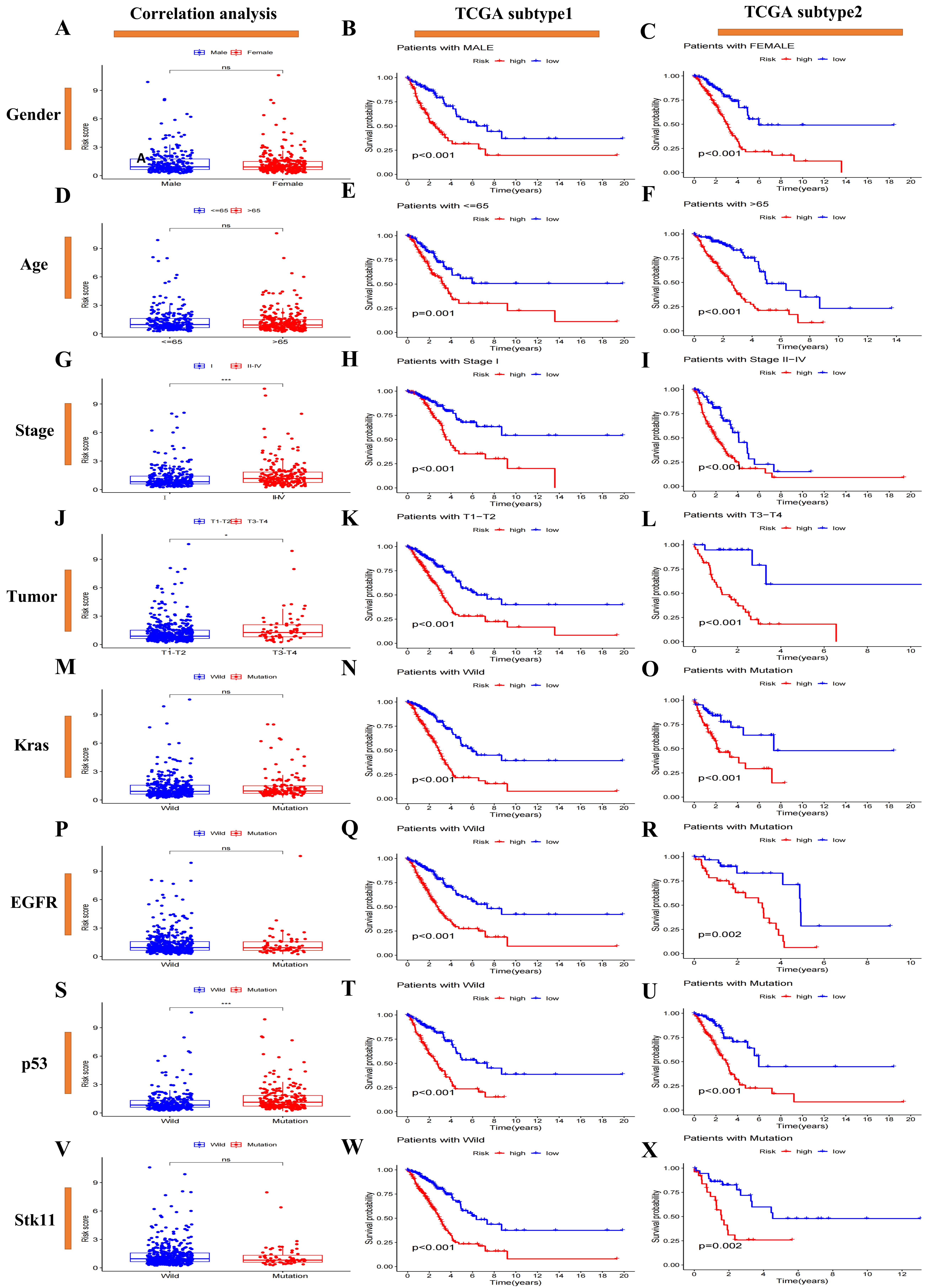

Supplement: Supplementary file 4 [file Image_4.pdf]
